# The real-world analysis of adverse events with teduglutide: a pharmacovigilance study based on the FAERS database

**DOI:** 10.3389/fphar.2024.1404658

**Published:** 2024-09-12

**Authors:** Xiaogan Wang, Hao Chen, Shuangshuang Han, Lingbo Li, Hongjin Chen, Bolin Yang

**Affiliations:** ^1^ First Clinical Medical College, Nanjing University of Chinese Medicine, Nanjing, China; ^2^ Inflammatory Bowel Disease Center/Department of Colorectal Surgery, Jiangsu Province Hospital of Chinese Medicine, Affiliated Hospital of Nanjing University of Chinese Medicine, Nanjing, China

**Keywords:** teduglutide, glucagon-like peptide 2, adverse drug events, disproportionality analysis, pharmacovigilance

## Abstract

**Background:**

Teduglutide, the first glucagon-like peptide 2 analogue, has been demonstrated to facilitate the absorption of gut nutrient and lessen the need for parenteral assistance in patients with Short Bowel Syndrome (SBS). However, its adverse drug events (AEs) are primarily documented in clinical trials, with a deficit in real-world data. This study evaluates the AEs profile of teduglutide based on Food and Drug Administration (FDA) Adverse Event Reporting System (FAERS) data.

**Method:**

A disproportionality analysis of FAERS data from Quarter 1 (Q1) 2013 to Quarter 3 (Q3) 2023 was conducted to examine the association between teduglutide and adverse events, employing Reporting Odds Ratio (ROR), Proportional Reporting Ratio (PRR), Bayesian Confidence Propagation Neural Network (BCPNN), and Empirical Bayesian Geometric Mean (EBGM) methods.

**Results:**

Out of 13,809,302 reports in the FAERS database, 10,114 reports identified teduglutide as the “primary suspect” in AEs identification. During the dosing observation period, the median occurrence of adverse events was 393 days (interquartile range [IQR] 97–996 days). Teduglutide-associated AEs occurred in 27 System Organ Classes (SOC), of which renal and urinary disorders is not mentioned in the specification. Based on the four algorithms, a total of 260 major disproportionality preferred terms (PTs) were filtered out, including previously unreported AEs including weight decreased (n = 805), vascular device infection (n = 683), dehydration (n = 596) and nephrolithiasis (n = 146).

**Conclusion:**

Our findings corroborate the AEs listed in the teduglutide prescribing information and additionally unveil new adverse reaction signals such as nephrolithiasis. These discoveries could aid in clinical monitoring and risk identification for teduglutide.

## 1 Introduction

Short Bowel Syndrome (SBS) is primarily a disorder in which the intestine’s functional length is less than 200 cm. It often results from intestinal surgical resections due to conditions like Crohn’s disease, traumatic strictures, mesenteric vascular complications and neoplasms, leading to a significant reduction in the absorption of intestinal nutrients (Pizzoferrato et al., 2022). Individuals with SBS-induced chronic intestinal failure (SBS-IF) fail to absorb the minimum required nutrients, necessitating intravenous supplementation of fluids, electrolytes, and nutrients for survival (Billiauws et al., 2018; Pironi et al., 2016). Parenteral nutrition (PN) remains the cornerstone of SBS management, but patients still face substantial challenges due to its associated complications like infections, septicemia, thrombus, and metabolic disorders along with diminished quality of life and elevated healthcare costs. In pursuit of a targeted therapy to enhance the absorptive capacity of the remaining intestine, several intestinal peptides have been investigated (Drucker et al., 2017; Vipperla and O'Keefe, 2014). Among these, GLP-2 has emerged as a promising candidate.

GLP-2 (glucagon-like peptide-2) is secreted by L-cells in the distal ileum and right colon in response to postprandial stimuli and has enterotrophic effects that promote absorption. Other peptides such as glucagon, GLP-1 (glucagon-like peptide-1) and glucose-dependent proinsulinotropic polypeptide are structurally similar to GLP-2, but GLP-2R (glucagon-like peptide-2 receptor) specifically recognises only GLP-2. (Jeppesen et al., 2001). The very short half-life of natural GLP-2 has been extended by subcutaneous injection of GLP-2 analogues. Teduglutide, the initial GLP-2 analogue approved by the FDA in 2012 to treat cases of SBS, improves intestinal absorption and mitigates diarrhea symptoms. It enhances intestinal adaptability and lessens the requirement for parenteral support, making it a popular treatment for SBS patients who are chronically reliant on parenteral nutrition. With the widespread adoption of Teduglutide, the medical management approach to SBS has transitioned from supportive therapy to more targeted treatment. In a double-blind, multicenter, placebo-controlled trial, the main goal was a reduction of more than 20% in the volume of weekly PN after 20 and 24 weeks of medication. Among the patients, 63% (27/43) receiving teduglutide at 0.05 mg/kg/day achieved the main goal, compared to 30% (13/43) of those getting placebo (*p* = 0.002) (Jeppesen et al., 2012). The Phase 3 Study of Teduglutide’s Effectiveness (STEPS) trial marked the initial evaluation of the relationship between PN volume reduction and improvements in Short Bowel Syndrome-Quality of Life (SBS-QoL). This trial revealed a statistically significant enhancement in SBS-QoL scores among patients treated with teduglutide at week 24 (Jeppesen et al., 2013). Therefore, teduglutide emerged as a pivotal intervention for augmenting the quality of life among individuals with SBS, showcasing its indispensable role compared to conventional supportive therapy in the aforementioned study.

Teduglutide is generally considered safe and reliable, with the most frequent side effects being abdominal pain, abdominal distension, and nasopharyngitis (Pape et al., 2020). However, the data mentioned above are major from clinical trails, and there may be a population selection gap between the incidence of adverse events (AEs) in the real world and the results of clinical trials with strict inclusion and exclusion criteria. Systematic studies based on real-world post-marketing data are necessary to provide a deeper comprehension of the AEs profile of teduglutide in clinical applications. The FAERS database, being the largest open drug vigilance database in the world, contains detailed medication information for all marketed drugs in the United States and has a broad demographic data of medication users. Unlike the AEs literature of drugs included in PubMed, EMBASE, and MEDLINE databases, the AEs information in the FAERS database is recorded and queried on the basis of individual cases, which makes the data more primitive. Furthermore, the FAERS database undergoes regular updates and is readily accessible to the public on the FDA’s official website, facilitating in-depth analysis to uncover emerging signals of AEs. At present, numerous research endeavors have tapped into the wealth of data within the FAERS database to scrutinize the AEs associated with clinical drug usage (Cirmi et al., 2020; Yu et al., 2021). This study comprehensively analyses the FAERS database and investigates teduglutide-related AEs associated with teduglutide, delving into the potential safety signals of the drug in real clinical practice to provide a basis for guiding clinical medication use safely. It is worth noting that much of the original information on AEs was self-reported by patients, which may introduce some bias into the results of related studies.

## 2 Method

### 2.1 Data source

As the FAERS database does not allow for searches by drug code, we conducted a comprehensive search for AEs linked to TEDUGLUTIDE, using “TEDUGLUTIDE”, “REVESTIVE” and “GATTEX” as keywords. This data set encompasses all relevant AEs from the first quarter of 2013 through the third quarter of 2023, due to teduglutide approved by the FDA in December 2012. To increase data accuracy and pinpoint the most direct relationship between the target drug and AEs, our analysis focused exclusively on AEs data where TEDUGLUTIDE was identified as the “PS (primary suspect)” drug. We further detected duplicate reports corresponding to AEs of the same drug for different case numbers by screening key fields such as age, gender, country and date of adverse events. These suspected duplicate reports were then excluded.

### 2.2 Data processing and analysis

From the DEMO table, we extracted PRIMARYID, CASEID, and FDA_DT fields, selecting the most recent report for each CASEID according to FDA guidance by sifting the entry with the maximal FDA_DT value. In instances of identical CASEID and FDA_DT, the report with the highest PRIMARYID was retained. Adverse event terminologies were normalized to the preferred terms (PTs) using the Medical Dictionary for Regulatory Activities (MedDRA) version 26.0, facilitating consistency and comparability. AEs were categorized and delineated depending on the System Organ Class (SOC) for a structured overview of AE characteristics. Post data acquisition, the clinical aspects of the reports, including demographics (gender, age), reporting country, year, and outcomes were analyzed. Hospitalization, disability, life-threatening conditions, and death were regarded as serious outcomes. A target reports/non-reports study employing a disproportionality approach was then conducted to identify signals for teduglutide at both PT and SOC levels, in comparison to other medications, using R software version 4.2.0 and Microsoft EXCEL 2019.

### 2.3 Statistical methods

The study employed a target reports/non-reports study disproportionality approach, widely used based on the principle of a 2 × 2 contingency table. If AEs with nausea were considered to be target reports (exposed group), then all other reports were considered to be non-report (unexposed group), including reports with other gastrointestinal events. Detailed calculation method is shown in Supplementary Table S1. An association between teduglutide and specific AEs was inferred when the proportion of AEs in the exposed group (reports) surpassed that in the unexposed group (non-reports), thereby indicating a disproportionality signal. A greater signal value indicates a stronger signal when it surpasses the threshold. To enhance the credibility of the data results and minimize the possibility of false positives, we performed statistical calculations on AEs with at least five reports, using the basic algorithms of Reporting Odds Ratio (ROR) (Rothman et al., 2004), and Proportional Reporting Ratio (PRR) (Evans et al., 2001). In addition the Bayesian algorithms of Bayesian Confidence Propagation Neural Network (BCPNN) (Bate et al., 1998) and Empirical Bayesian Geometric Mean (EBGM) (Dumouchel, 1999) also were used. The specific algorithms and positive threshold signal determination standards were implemented based on previous literature methods, with further details provided in Supplementary Table S2. Instead, the correlation between teduglutide and AEs was considered only if all four algorithms were satisfied simultaneously.

## 3 Results

### 3.1 Demographic characteristics of AEs

**FIGURE 1:**
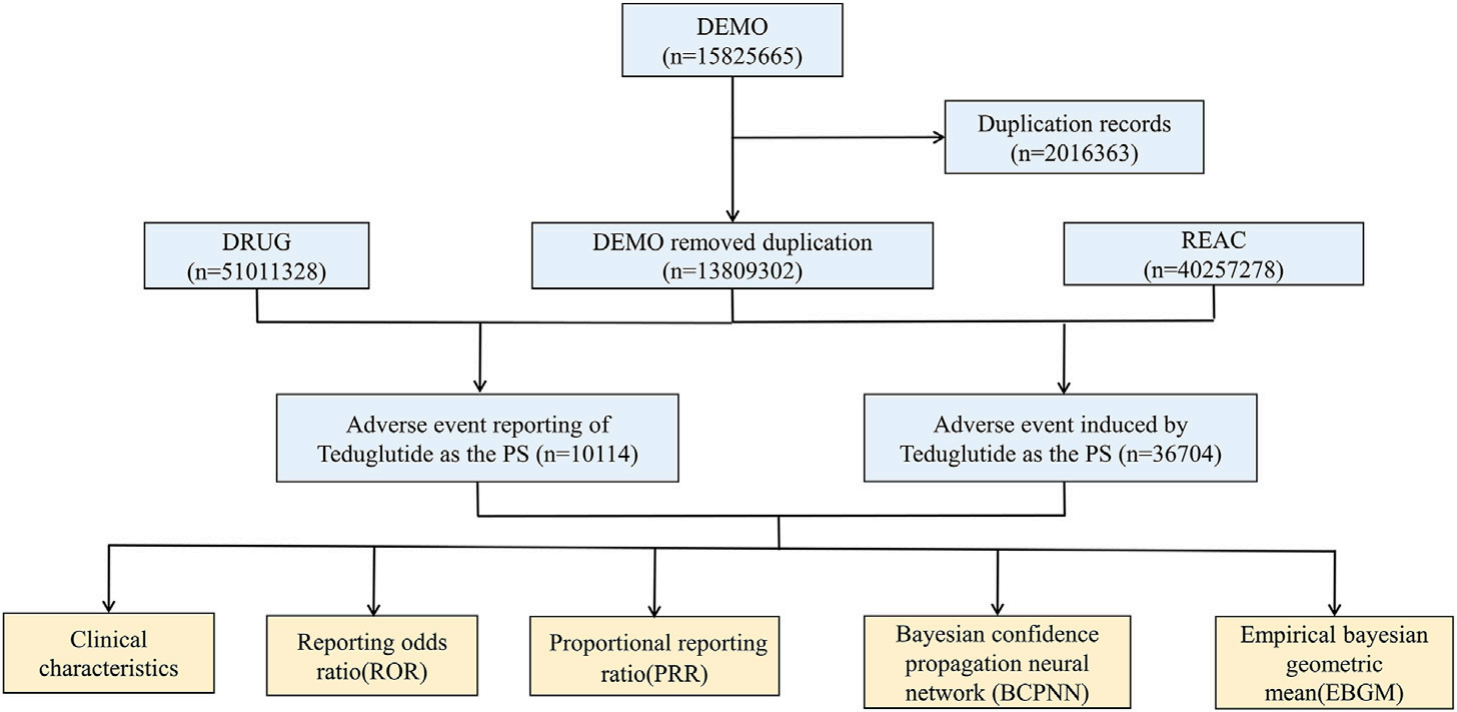
Flow chart of selecting reports of teduglutide-related AEs from the FAERS database.

**FIGURE 2:**
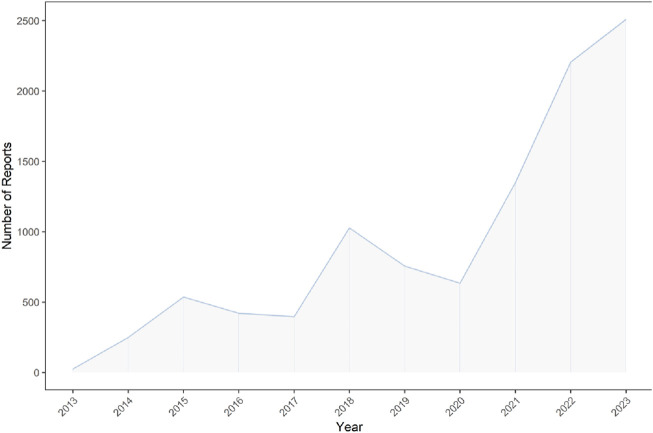
Reports number and trends of teduglutide-related AEs.

Our analysis of the FAERS database yielded 40,257,878 reports from January 2013 to September 2023. Post data cleaning, 10,114 reports were deemed suitable for in-depth analysis. Details of the data collection, interpreting and analysis process are shown in Figure 1, which shows that 10,114 patients reported 36,704 adverse reactions. The baseline patient information corresponding to the final screening of adverse event reports is shown in Table 1. Among the identified teduglutide AEs, females predominated with 5,715 (56.51%). Regarding age distribution, an unspecified age group accounted for a significant portion (4,291 reports, 42.43%), but from the known data, the majority of AEs occurred in the group aged 45–64 (2,382 reports, 23.55%). Reporters were primarily consumers (4,081 reports, 40.40%), followed by pharmacists (3,240, 32.00%) and physicians (1,600 reports, 15.82%). Among the countries reporting AEs, the greatest quantity of reports was from the United States (7,350 reports, 72.67%), followed by Germany (720 reports, 7.12%) and France (666 reports, 6.58%). As Figure2 shows, a continuous increase in reported AEs was noted since the drug’s market introduction, with the majority between 2021 and 2023 (6,067 reports, 59.99%). Clinically, serious adverse outcomes associated with the drug since its launch primarily included hospitalization, disability, life-threatening conditions, and death, with hospitalization being the most frequent (5,283 reports, 37.11%), followed by death (703 reports, 4.94%). Our data showed that the median adverse event occurrence time during the observation period was 393 days (interquartile range [IQR] 97–996 days). Most of the AEs (1871reports, 49.80%) occurred after 1 year of dosing followed by 1 month after dosing (511reports, 13.62%). The lowest number of AEs was found in 61–90 days (137reports, 3.65%).

**TABLE 1:** Clinical character of reports associated with teduglutide (n = 10114).

Characters	Number of reports (%)
Gender	
Female	5,715 (56.51%)
Male	3,459 (34.20%)
Unknown	940 (9.29%)
Age	
<18	819 (8.10%)
18–44	866 (8.56%)
45–64	2,382 (23.55%)
65–74	1,232 (12.18%)
≥75	524 (5.18%)
Unknown	4,291 (42.43%)
Reporter	
Consumer	4,081 (40.35%)
Pharmacist	260 (2.57%)
Physician	1,600 (15.82%)
Other health professionals	4,015 (39.70%)
Unknown	185 (1.83%)
Occurrence Country	
United states	7,350 (72.67%)
Canada	222 (2.19%)
Japan	256 (2.53%)
Germany	720 (7.12%)
France	666 (6.58%)
Other	900 (8.90%)
Serious outcome	
Death	703 (4.94%)
Disability	62 (0.44%)
Hospitalization	5,283 (37.11%)
Life-threatening	142 (1.00%)
AEs Time - Medication Date(days)	
0–30	511 (13.62%)
31–60	215 (5.73%)
61–90	137 (3.65%)
91–180	373 (9.93%)
181–360	465 (12.38%)
>360	1871 (49.80%)

### 3.2 Signal detection result

Due to the inability of AEs at the SOC level to simultaneously meet the requirements of all four algorithms, we defined a signal indicator as significant when calculating teduglutide-related AEs at the SOC level if it met any one of the four algorithms. As shown in Table 2, all AEs of the drug encompassed a total of 27 organ systems, with the high signal SOC associated with teduglutide concentrated in the following categories: gastrointestinal disorders (n = 7,199, ROR = 2.66, PRR = 2.34, EBGM = 2.33, IC = 1.22, *P*< 0.001), infections and infestations (n = 4,789, ROR = 2.68, PRR = 2.68, EBGM = 2.45, IC = 1.30, *P*< 0.001), metabolism and nutrition disorders (n = 2,090, ROR = 2.86, PRR = 2.75, EBGM = 2.75, IC = 1.46, *P*< 0.001), renal and urinary disorders (n = 1,025, ROR = 1.46, PRR = 1.45, EBGM = 1.45, IC = 0.53, *P*< 0.001), and hepatobiliary disorders (n = 493, ROR = 1.66, PRR = 1.65, EBGM = 1.65, IC = 0.72, *P*< 0.001). Metabolism and nutrition disorders presented the highest signal, while gastrointestinal disorders were the most frequently reported SOC. Notably, renal and urinary disorders emerged as a new SOC signal not previously highlighted in teduglutide prescribing information.

**TABLE 2:** The signal strength of reports associated with teduglutide at the SOC level.

Soc	Report	ROR (95%CI)	PRR (95%CI)	EBGM (EBGM05)	$x^{2}$	IC (IC025)	Adjusted P
Gastrointestinal disorders	7,199	2.66 (2.59–2.73)	2.34 (2.28–2.4)	2.33 (2.27)	5,989.27	1.22 (−0.44)	<0.001^*^
General disorders and administration site conditions	5,407	0.79 (0.77–0.81)	0.82 (0.8–0.84)	0.82 (0.80)	262.29	−0.29 (−1.95)	<0.001^*^
Infections and infestations	4,789	2.68 (2.6–2.76)	2.46 (2.38–2.53)	2.45 (2.38)	4,361.35	1.30 (−0.37)	<0.001^*^
Investigations	3,250	1.58 (1.52–1.63)	1.52 (1.47–1.58)	1.52 (1.47)	622.73	0.61 (−1.06)	<0.001^*^
Metabolism and nutrition disorders	2090	2.86 (2.73–2.99)	2.75 (2.63–2.88)	2.75 (2.63)	2,373.27	1.46 (−0.21)	<0.001^*^
Surgical and medical procedures	1,274	2.62 (2.48–2.77)	2.57 (2.43–2.71)	2.56 (2.42)	1,231.1	1.36 (−0.31)	<0.001^*^
Renal and urinary disorders	1,025	1.46 (1.37–1.55)	1.45 (1.36–1.54)	1.45 (1.36)	143.83	0.53 (−1.13)	<0.001^*^
Product issues	686	1.11 (1.02–1.19)	1.10 (1.02–1.19)	1.10 (1.02)	6.77	0.14 (−1.52)	0.27
Hepatobiliary disorders	493	1.66 (1.51–1.81)	1.65 (1.51–1.8)	1.65 (1.51)	126.08	0.72 (−0.95)	<0.001^*^
Social circumstances	196	1.23 (1.07–1.42)	1.23 (1.07–1.42)	1.23 (1.07)	8.71	0.3 (−1.36)	0.10

Adjusted p: Bonferroni-corrected *p*-values.

*Adjusted *p*-value < 0.001 were considered statistically significant.

**FIGURE 3:**
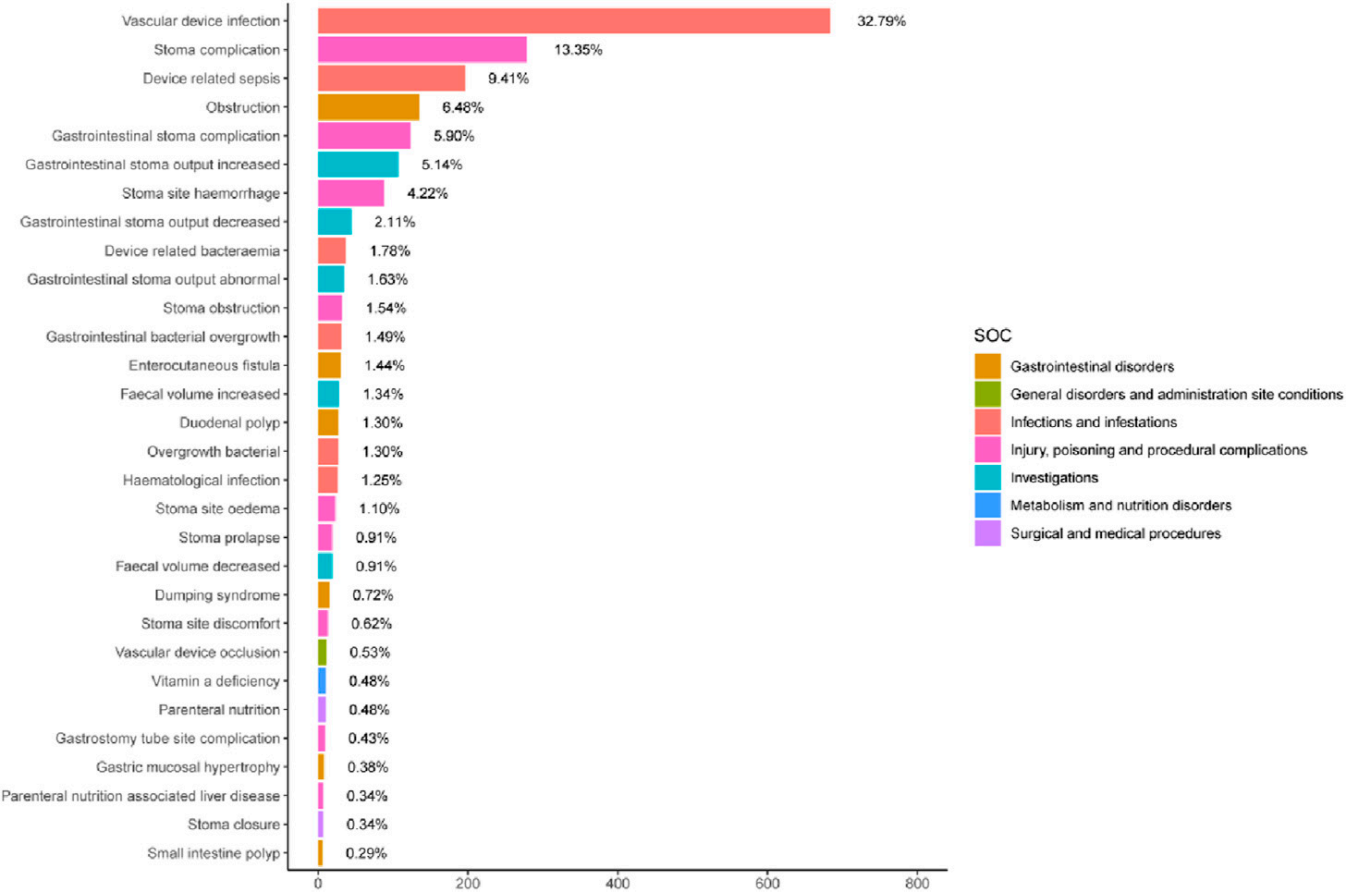
The top 30 signal strength of teduglutide-related PTs sorted by EBGM signal at the PT level. The color represents the SOC corresponding to the PT, and the percentage value represents the ratio of the occurrence of PT to the sum of the top 30 signal strength.

Supplementary Table S3 showed 260 PTs that met all four algorithm criteria at the PT level. In the table we can find abdominal pain (n = 773, EBGM = 5.80) and abdominal distension (n = 482, EBGM = 7.99), which were in line with the common AEs in the pharmaceutical instructions. These 260 PTs were ranked according to report number and the top 30 PTs in terms of number of reports were selected for inclusion in Table 3, which showed that the top 5 PTs in terms of number of morbidities were weight decreased (n = 805, EBGM = 4.86), abdominal pain (n = 773, EBGM = 5.80), vascular device infection (n = 683, EBGM = 257.96), hospitalisation (n = 623, EBGM = 6.45), and dehydration (n = 596, EBGM = 8.01). Moreover, we ranked the PTs in Supplementary Table S3 according to the strength of the EBGM algorithm, and finally obtained the top 30 PTs in terms of signal strength to be included in Table 4. The results showed that the top 5 in terms of signal strength were all related to stoma, which were stoma prolapse (n = 19, EBGM = 548.41), gastrointestinal stoma output decreased (n = 44, EBGM = 530.33), gastrointestinal stoma output abnormal (n = 34, EBGM = 484.31), gastrointestinal stoma output increased (n = 107, EBGM = 467.56), stoma complication (n = 278, EBGM = 412.04). Figure 3 showed the top 30 PTs by signal strength, categorized by SOC level, with the largest proportion attributed to infections and infestations. In addition to the common AEs explicitly mentioned with the specification, we also identified suspected AEs not mentioned in the specification, such as weight decreased (n = 805, EBGM = 4.86), vascular device infection (n = 683, EBGM = 257.96), dehydration (n = 596, EBGM = 8.01), device related infection (n = 448, EBGM = 42.93) and nephrolithiasis (n = 146, EBGM = 5.43). Other unexpected PTs in drug instructions were displayed in Supplementary Table S3.

**TABLE 3:** The top 30 PTs of teduglutide selected based on a level of 260 PTs that met the four algorithmic criteria.

soc	PT	Case	ROR (95%CI)	PRR (95%CI)	EBGM (EBGM05)	$x^{2}$	IC (IC025)	Adjusted P
Investigations	Weight decreased	805	4.96 (4.63–5.32)	4.88 (4.55–5.23)	4.86 (4.58)	2,480.74	2.28 (0.61)	<0.001^*^
Investigations	Weight increased	564	4.45 (4.1–4.84)	4.40 (4.05–4.78)	4.38 (4.09)	1,480.09	2.13 (0.47)	<0.001^*^
Investigations	Gastrointestinal stoma output increased	107	816.62 (635.75–1,048.96)	814.25 (633.9–1,045.9)	467.56 (379.19)	49861.34	8.87 (7.19)	<0.001^*^
Investigations	Product availability issue	105	9.19 (7.59–11.14)	9.17 (7.57–11.12)	9.10 (7.75)	758.23	3.19 (1.52)	<0.001^*^
Investigations	Blood magnesium decreased	78	15.49 (12.38–19.37)	15.46 (12.36–19.33)	15.26 (12.65)	1,040.32	3.93 (2.27)	<0.001*
Gastrointestinal disorders	Abdominal pain	773	5.93 (5.52–6.37)	5.83 (5.43–6.26)	5.80 (5.46)	3,085.51	2.54 (0.87)	<0.001^*^
Gastrointestinal disorders	Abdominal distension	482	8.14 (7.43–8.9)	8.04 (7.35–8.8)	7.99 (7.41)	2,955.08	3.00 (1.33)	<0.001^*^
Gastrointestinal disorders	Flatulence	247	7.89 (6.96–8.95)	7.85 (6.92–8.9)	7.80 (7.02)	1,466.44	2.96 (1.30)	<0.001^*^
Gastrointestinal disorders	Crohn’s disease	180	4.66 (4.02–5.39)	4.64 (4.01–5.37)	4.62 (4.09)	512.42	2.21 (0.54)	<0.001^*^
Gastrointestinal disorders	Obstruction	135	64.92 (54.56–77.25)	64.68 (54.36–76.97)	61.14 (52.86)	7,993.21	5.93 (4.27)	<0.001^*^
Gastrointestinal disorders	Abnormal faeces	112	22.90 (18.99–27.62)	22.83 (18.93–27.54)	22.39 (19.14)	2,290.85	4.48 (2.82)	<0.001^*^
Gastrointestinal disorders	Hypervolaemia	94	7.14 (5.83–8.75)	7.13 (5.82–8.73)	7.09 (5.98)	492.15	2.83 (1.16)	<0.001^*^
Gastroinestinal disorders	Hypokalaemia	89	3.52 (2.86–4.33)	3.51 (2.85–4.33)	3.50 (2.94)	159.50	1.81 (0.14)	<0.001^*^
Gastrointestinal disorders	Frequent bowel movements	83	5.3 (4.27–6.58)	5.29 (4.27–6.57)	5.27 (4.40)	287.79	2.40 (0.73)	<0.001^*^
Infections and infestations	Vascular device infection	683	343.35 (314.94–374.33)	336.98 (309.1–367.38)	257.96 (239.98)	174996.59	8.01 (6.34)	<0.001^*^
Infections and infestations	Device related infection	448	45.17 (41.08–49.68)	44.63 (40.59–49.08)	42.93 (39.64)	18367.11	5.42 (3.76)	<0.001^*^
Infections and infestations	Sepsis	369	5.82 (5.25–6.45)	5.77 (5.21–6.39)	5.74 (5.27)	1,449.60	2.52 (0.86)	<0.001^*^
Infections and infestations	Staphylococcal infection	92	5.18 (4.22–6.35)	5.17 (4.21–6.34)	5.15 (4.33)	307.73	2.36 (0.70)	<0.001^*^
Surgical and medical procedures	Hospitalisation	623	6.58 (6.07–7.12)	6.48 (5.99–7.02)	6.45 (6.03)	2,878.20	2.69 (1.02)	<0.001^*^
Surgical and medical procedures	Therapy interrupted	210	6.60 (5.76–7.56)	6.56 (5.73–7.52)	6.53 (5.83)	985.50	2.71 (1.04)	<0.001^*^
Surgical and medical procedures	Weight fluctuation	91	13.67 (11.11–16.81)	13.64 (11.09–16.78)	13.48 (11.34)	1,052.96	3.75 (2.09)	<0.001^*^
Metabolism and nutrition disorders	Dehydration	596	8.17 (7.54–8.87)	8.06 (7.43–8.74)	8.01 (7.48)	3,664.67	3.00 (1.34)	<0.001^*^
Metabolism and nutrition disorders	Fluid retention	110	3.36 (2.79–4.05)	3.35 (2.78–4.05)	3.35 (2.86)	181.36	1.74 (0.08)	<0.001^*^
Metabolism and nutrition disorders	Fistula	90	14.22 (11.55–17.51)	14.19 (4.36–6.59)	14.02 (11.78)	1,089.20	3.81 (2.14)	<0.001^*^
Metabolism and nutrition disorders	Malnutrition	79	13.79 (11.05–17.23)	13.77 (11.02–17.19)	13.61 (11.30)	923.89	3.77 (2.10)	<0.001^*^
Injury, poisoning and procedural complications	Stoma complication	278	664.41 (572.36–771.26)	659.38 (568.03–765.42)	412.04 (363.71)	114099.16	8.69 (7.02)	<0.001^*^
Injury, poisoning and procedural complications	Gastrointestinal stoma complication	123	139.13 (115.3–167.88)	138.67 (114.92–167.32)	123.2 (105.28)	14923.00	6.94 (5.28)	<0.001^*^
Renal and urinary disorders	Nephrolithiasis	146	5.47 (4.65–6.44)	5.45 (4.63–6.42)	5.43 (4.74)	528.63	2.44 (0.78)	<0.001^*^
General disorders and administration site conditions	Complication associated with device	111	8.11 (6.73–9.78)	8.09 (6.71–9.75)	8.04 (6.87)	685.04	3.01 (1.34)	<0.001^*^
Hepatobiliary disorders	Cholelithiasis	88	5.65 (4.58–6.96)	5.64 (4.57–6.95)	5.61 (4.71)	333.97	2.49 (0.82)	<0.001^*^

Adjusted p: Bonferroni-corrected *p*-values.

*Adjusted *p*-value < 0.001 were considered statistically significant.

**TABLE 4:** The top 30 signal strength of teduglutide-related AEs sorted by the MGPS algorithm (EBGM05 > 2) at the PT level.

soc	PT	Case	ROR (95%CI)	PRR (95%CI)	EBGM (EBGM05)	$x^{2}$	IC (IC025)	Adjusted P
Injury, poisoning and procedural complications	Stoma prolapse	19	1,096.38 (580.43–2070.94)	814.25 (633.9–1,045.9)	548.40 (322.10)	10391.20	9.10 (7.37)	<0.001^*^
Injury, poisoning and procedural complications	Stoma complication	278	664.41 (572.36–771.26)	1,095.81 (580.13–2069.86)	412.04 (363.71)	114099.16	8.69 (7.02)	<0.001^*^
Injury, poisoning and procedural complications	Stoma obstruction	32	265.88 (180.68–391.27)	266.55 (175.01–405.96)	214.01 (154.9)	6790.73699	7.74 (6.06)	<0.001^*^
Injury, poisoning and procedural complications	Stoma site oedema	23	240.18 (152.94–377.19)	225.85 (149.23–341.81)	197.08 (135.09)	4,491.13	7.62 (5.94)	<0.001^*^
Injury, poisoning and procedural complications	Parenteral nutrition associated liver disease	7	109.60 (50.39–238.39)	117.83 (61.37–226.24)	99.71 (52.04)	684.66	6.64 (4.94)	<0.001^*^
Injury, poisoning and procedural complications	Gastrointestinal stoma complication	123	139.13 (115.3–167.88)	138.67 (114.92–167.32)	123.20 (105.28)	14923.00	6.94 (5.28)	<0.001^*^
Injury, poisoning and procedural complications	Stoma site haemorrhage	88	87.63 (70.51–108.91)	86.03 (53.93–137.25)	81.04 (67.56)	6963.20	6.34 (4.67)	<0.001^*^
Injury, poisoning and procedural complications	Stoma site discomfort	13	83.83 (47.69–147.36)	109.58 (50.38–238.34)	77.92 (48.6)	987.97	6.28 (4.60)	<0.001^*^
Injury, poisoning and procedural complications	Gastrostomy tube site complication	9	73.62 (37.48–144.59)	83.80 (47.67–147.30)	69.03 (39.24)	603.95	6.11 (4.43)	<0.001^*^
Investigations	Gastrointestinal stoma output decreased	44	1,027.09 (680.76–1,549.62)	1,025.86 (679.94–1,547.77)	530.33 (375.91)	23267.66	9.05 (7.36)	<0.001^*^
Investigations	Gastrointestinal stoma output abnormal	34	867.26 (553.03–1,360.03)	659.38 (568.03–765.42)	484.31 (332.37)	16413.45	8.92 (7.22)	<0.001^*^
Investigations	Gastrointestinal stoma output increased	107	816.62 (635.75–1,048.96)	866.45 (552.52–1,358.77)	467.56 (379.19)	49861.34	8.87 (7.19)	<0.001^*^
Investigations	Faecal volume increased	28	100.35 (68.14–147.78)	87.35 (61.27–124.53)	91.95 (66.51)	2,521.17	6.52 (4.85)	<0.001^*^
Investigations	Faecal volume decreased	19	86.08 (53.96–137.32)	87.90 (51.94–148.75)	79.84 (54.01)	1,480.64	6.32 (4.64)	<0.001^*^
Infections and infestations	Vascular device infection	683	343.35 (314.94–374.33)	336.98 (309.1–367.38)	257.96 (239.98)	174996.59	8.01 (6.34)	<0.001^*^
Infections and infestations	Device related bacteraemia	37	279.90 (195.06–401.65)	279.62 (194.86–401.25)	222.98 (164.83)	8,183.83	7.80 (6.12)	<0.001^*^
Infections and infestations	Overgrowth bacterial	27	226.02 (149.34–342.07)	165.98 (142.78–192.95)	187.43 (132.51)	5,011.32	7.55 (5.87)	<0.001^*^
Infections and infestations	Device related sepsis	196	166.87 (143.54–193.98)	249.05 (125.33–494.90)	144.28 (127.20)	27914.37	7.17 (5.51)	<0.001^*^
Infections and infestations	Gastrointestinal bacterial overgrowth	31	58.62 (40.84–84.14)	73.60 (37.47–144.55)	55.65 (41.12)	1,665.20	5.80 (4.13)	<0.001^*^
Infections and infestations	Haematological infection	26	51.37 (34.66–76.13)	51.34 (34.64–76.08)	49.08 (35.32)	1,225.81	5.62 (3.95)	<0.001^*^
Metabolism and nutrition disorders	Vitamin a deficiency	10	249.12 (125.36–495.03)	240.03 (152.85–376.96)	203.11 (114.34)	2013.06	7.67 (5.96)	<0.001^*^
Gastrointestinal disorders	Duodenal polyp	27	266.74 (175.14–406.26)	265.65 (180.52–390.93)	214.59 (150.92)	5,745.40	7.75 (6.06)	<0.001^*^
Gastrointestinal disorders	Small intestine polyp	6	126.46 (54.31–294.44)	87.43 (70.35–108.65)	113.46 (55.94)	669.44	6.83 (5.12)	<0.001^*^
Gastrointestinal disorders	Enterocutaneous fistula	30	64.89 (44.89–93.8)	58.57 (40.8–84.07)	61.27 (45.02)	1780.36	5.94 (4.27)	<0.001^*^
Gastrointestinal disorders	Obstruction	135	64.92 (54.56–77.25)	70.49 (38.31–129.70)	61.14 (52.86)	7,993.22	5.93 (4.27)	<0.001^*^
Gastrointestinal disorders	Gastric mucosal hypertrophy	8	63.54 (31.15–129.61)	44.63 (40.59–49.08)	60.10 (33.10)	465.35	5.91 (4.23)	<0.001^*^
Gastrointestinal disorders	Dumping syndrome	15	87.93 (51.96–148.81)	126.44 (54.31–294.39)	81.45 (52.44)	1,192.97	6.35 (4.67)	<0.001^*^
General disorders and administration site conditions	Vascular device occlusion	11	70.51 (38.32–129.74)	64.84 (44.86–93.72)	66.29 (39.80)	708.01	6.05 (4.37)	<0.001^*^
General disorders and administration site conditions	Catheter site inflammation	16	42.68 (25.9–70.33)	42.66 (2.47–6.61)	41.1 (27.06)	626.54	5.36 (3.69)	<0.001^*^
Surgical and medical procedures	Parenteral nutrition	10	117.86 (61.38–226.30)	100.27 (68.08–147.67)	106.49 (61.69)	1,045.91	6.73 (5.04)	<0.001^*^
Surgical and medical procedures	Stoma closure	7	54.80 (25.65–117.08)	49.88 (34.8–71.51)	52.23 (27.67)	352.06	5.71 (4.02)	<0.001^*^

Adjusted p: Bonferroni-corrected *p*-values.

*Adjusted *p*-value < 0.001 were considered statistically significant.

### 3.3 Serious vs. non-serious reports

The top 30 PTs by number of reports were selected for comparison between serious and non-serious outcomes, as shown in Table 5. In terms of gender, females was more likely to have a serious adverse outcome than males (60.53% vs. 39.47%, *P*< 0.001). Regarding age, no significant difference was observed between the two types of outcomes in each age group. In terms of PTs, abdominal complaints such as abdominal pain and bloating mentioned in the specification were more likely to be non-serious outcome. Among the new AEs not mentioned in the specification, weight loss (8.48% vs. 8.87%, *p* = 0.573) and nephrolithiasis (1.45% vs. 1.49%, *p* = 0.946) did not show a significant difference between the two endpoints, while device related infection (9.28% vs. 3.48%, *P*< 0.001) and dehydration (7.61% vs. 3.64%, *P*< 0.001) tended to occur as serious adverse events.

**TABLE 5:** Differences in top 30 PTs of serious and non-serious reports.

	Serious cases	Non-serious cases	P
Gender			<0.001^*^
Female	3,266 (60.53%)	2,449 (64.82%)	
Male	2,130 (39.47%)	1,329 (35.18%)	
Age			0.131
<18	555 (14.90%)	264 (12.59%)	
18–44	536 (14.39%)	330 (15.74%)	
45–64	1,510 (40.53%)	872 (41.58%)	
65–74	807 (21.66%)	452 (21.55%)	
≥75	318 (8.53%)	179 (8.54%)	
Types of PTs	—	—	P
Weight decreased	449 (7.82%)	356 (8.14%)	0.573
Abdominal pain	409 (7.12%)	364 (8.33%)	0.026
Vascular device infection	531 (9.25%)	152 (3.48%)	<0.001^*^
Hospitalisation	623 (10.10%)	0 (0.00%)	<0.001^*^
Dehydration	437 (7.61%)	159 (3.64%)	<0.001^*^
Weight increased	273 (4.75%)	291 (6.66%)	<0.001^*^
Abdominal distension	210 (3.66%)	272 (6.22%)	<0.001^*^
Device related infection	330 (5.75%)	118 (2.70%)	<0.001^*^
Sepsis	308 (5.36%)	61 (1.40%)	<0.001^*^
Intestinal obstruction	201 (3.50%)	100 (2.29%)	<0.001^*^
Stoma complication	133 (2.32%)	145 (3.32%)	0.003
Flatulence	104 (1.81%)	143 (3.27%)	<0.001^*^
Therapy interrupted	149 (2.59%)	61 (1.40%)	<0.001^*^
Device related sepsis	163 (2.84%)	33 (0.75%)	<0.001^*^
Crohn’s disease	96 (1.67%)	84 (1.92%)	0.386
Nephrolithiasis	82 (1.43%)	64 (1.46%)	0.946
Obstruction	76 (1.32%)	59 (1.35%)	0.978
Gastrointestinal stoma complication	71 (1.24%)	52 (1.19%)	0.904
Abnormal faeces	53 (0.92%)	59 (1.35%)	0.053
Complication associated with device	86 (1.50%)	25 (0.57%)	<0.001^*^
Fluid retention	54 (0.94%)	56 (1.28%)	0.123
Gastrointestinal stoma output increased	62 (1.08%)	45 (1.03%)	0.884
Small intestinal obstruction	74 (1.29%)	32 (0.73%)	0.009
Product availability issue	63 (1.1%)	42 (0.96%)	0.569
Hypervolaemia	55 (0.96%)	39 (0.89%)	0.814
Staphylococcal infection	72 (1.25%)	20 (0.46%)	<0.001^*^
Weight fluctuation	41 (0.71%)	50 (1.14%)	0.031
Fistula	60 (1.04%)	30 (0.69%)	0.073
Blood potassium decreased	63 (1.10%)	27 (0.62%)	0.015
Hypokalaemia	48 (0.84%)	41 (0.94%)	0.662

P: pearson χ 2 test.

*p-value < 0.001 were considered statistically significant.

### 3.4 Pediatric patients-based difference in risk signals

**FIGURE 4:**
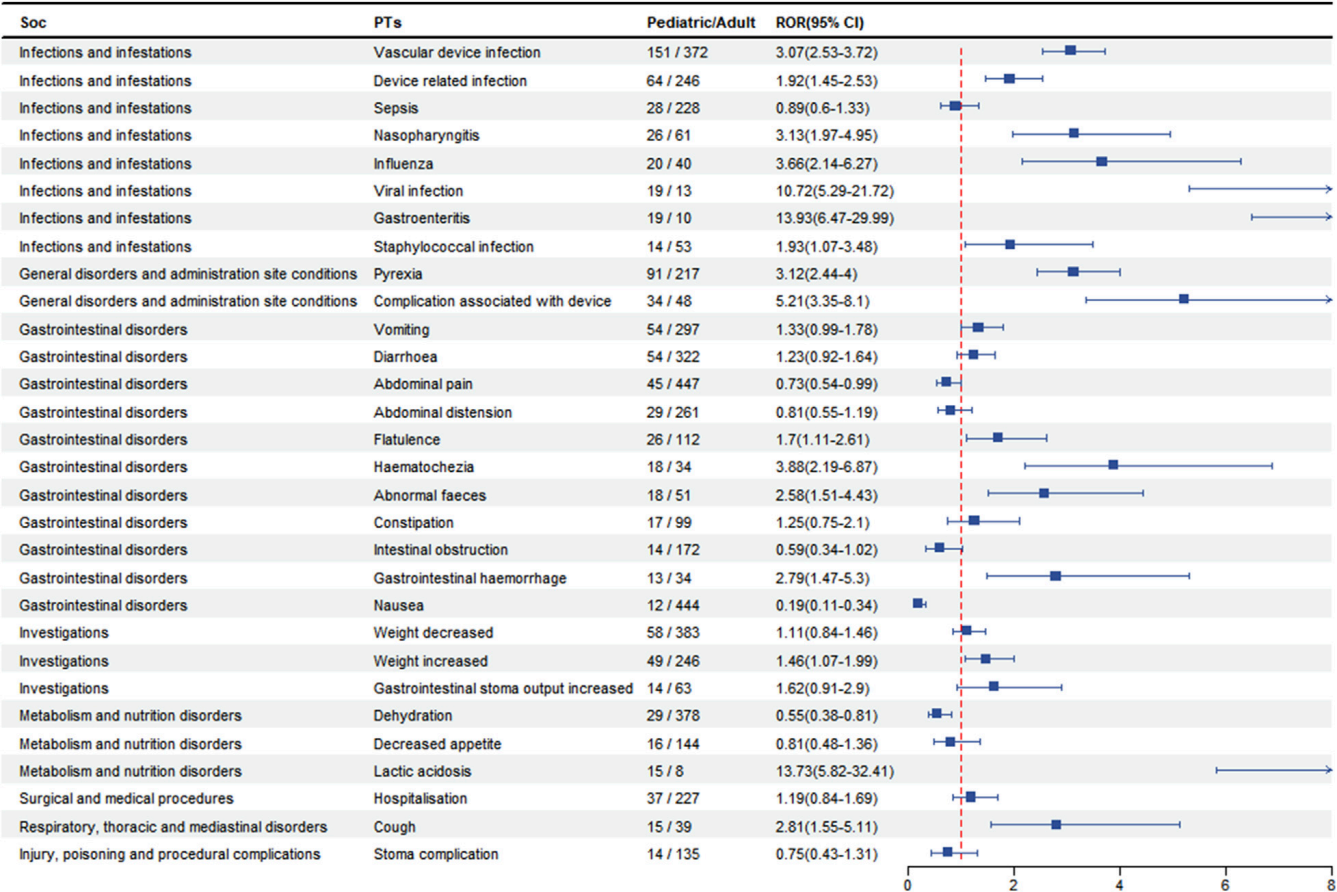
Analysis of pediatric-differentiated risk signals in teduglutide.

To analyse the pediatric patients (age <18 years) for AEs, we screened patients with a disproportionate incidence of AEs between in the top 30 PTs and categorised them by SOC. As shown in Figure 4, AEs in pediatric patients in the SOC were concentrated in infections and infestations, gastrointestinal disorders, and general disorders. In terms of AEs, we found that device infections (n = 151, 95% CI: 2.53–3.72) were the most prevalent, followed by pyrexia (n = 91, 95% CI: 2.44–4.00) and vomiting (n = 54, 95% CI: 0.99–1.78), which were more likely to occur in pediatric patients than in adults. However, the risk of was higher in the adult patient than in the pediatric patient for abnormal pian (n = 45, 95%CI: 0.54–0.99), nausea (n = 12, 95%CI: 0.19–0.11) and dehydration (n = 29, 95%CI: 0.38–0.81).

## 4 Discussion

Our study is the first to comprehensively document and assess the safety of post marketing administration of teduglutide using multiple algorithms based on FARES database to date. This aims to serve as a reference for future clinical medication safety. Teduglutide has been on the market for over a decade and is widely used in clinical practice. Our study has found a sustained increase in the number of AEs for this drug from after the first quarter of 2013 to the third quarter of 2023. Notably, nearly 60% of all AEs occurred in the last 3 years (6,067 reports), indicating that may be related to the surge in drug use in the last years. The continuing increase in adverse events and potential clinical risks needs to be taken seriously the need for ongoing epidemiological surveillance strongly emphasised. In terms of the sex ratio of patients, our findings show that females are higher than males (56.51%.vs. 34.20%). One of the possible explanations for this is the higher prevalence of SBS in females than in males (Siddiqui et al., 2021). Previous studies have shown that the apparent clearance of teduglutide in males is approximately 18% higher than in females (12.4 vs. 10.5 L per hour) (Marier et al., 2010), which also explains the results of our study. Furthermore, the majority of reports in study occurred in individuals aged 45–64 years old (23.55%), which may be associated with the age peak of disease onset. It is reported that SBS mainly occurs in middle-aged and elderly people, with 67.8% of patients being over 50 years old and an average age of 56.6 years (Siddiqui, Al-Yaman, Singh and Kirby, 2021), which is consistent with the results of our dataset analysis. The median time to onset of teduglutide-related adverse events was 396 days (interquartile range [IQR] 97–996 days), with the majority of events occurring after 1 year (1871 reports, 49.80%), suggesting that the number of adverse events may increase with long-term treatment.

AEs at the SOC level revealed gastrointestinal disorders as the most prevalent, a foreseeable outcome given the localization of glucagon-like peptide-2 receptor (GLP-2R) in the gastrointestinal tract. The common manifestations in the instructions and clinical reports are consistent with the frequently occurring AEs in this study’s gastrointestinal system, such as abdominal pain (n = 773, EBGM = 5.80) and abdominal distension (n = 482, EBGM = 7.99). These symptoms typically emerge early in medication use and gradually subside thereafter (Loutfy et al., 2022; Pape et al., 2020). Furthermore, metabolic and nutrition disorders emerged with the highest signal strength, indicating potential imbalances caused by enhanced intestinal absorption and modifications in parenteral nutrition strategy. (Jeppesen et al., 2012). Significantly, renal diseases and urinary disorders presented as a notable signal not previously emphasized in the prescribing information, warranting additional investigation into teduglutide’s renal effects. Studies have shown that GLP-2 is primarily degraded within the body by the exopeptidase dipeptidyl peptidase IV (DPP-IV), with the highest DDP-IV activity in the kidneys. After nephrectomy, rat’s circulation level of GLP-2 continues to increase, and research on the metabolism of the drug teduglutide has also confirmed the correlation between exposure and the degree of kidney damage (Drucker et al., 1997; Nave et al., 2013). Due to the lack of concomitant medication and clinical status of the patients, we cannot exclude the correlation of adverse renal function events with other conditions, so our results can only be regarded as indicative.

At the PT level, AEs not mentioned in the prescribing information include weight decrease, catheter infections, dehydration, and nephrolithiasis. The most frequently reported AEs was weight decrease (n = 805, EBGM = 4.86), which may relate to GLP-2 being an intermediary in the gut-brain axis of energy balance. Research has identified a neuron pathway containing GLP-2 that links the nucleus of the solitary tract and the dorsomedial hypothalamic nucleus (DMH), potentially exerting a suppressive effect on food intake. GLP-2-induced regulation of DMH on nutrition and weight could enhance glucose tolerance and insulin sensitivity, inhibit basal hepatic glucose production, and influence feeding behavior through effects on neuroendocrine and visceral information, possibly underlying the mechanism of weight decreased (Shi et al., 2013; Tang-Christensen et al., 2000). Previous studies suggest that teduglutide reduces fecal wet weight excretion and improves intestinal absorption, with its effects associated with an increase in lean body mass, but no change or even a decrease in fat mass has been observed (Jeppesen et al., 2011). This might also relate to the drug’s efficacy and adjustments in parenteral nutrition status, where the balance between oral intake and parenteral nutrition supply can affect weight fluctuations.

The occurrence of dehydration is primarily associated with the specificities of SBS patients, such as after ileostomy surgery at the end of the jejunum, where patients’ needs for energy, fluids, and electrolytes undergo significant changes. The loss of substantial amounts of sodium and water may pose a chronic dehydration risk (Pedersen et al., 2020). Patients with SBS exhibit considerable heterogeneity, similarly affecting the heterogeneity in responses to teduglutide. Drug’s ability to reduce dependency on parenteral nutrition—if excessive reduction in parenteral nutrition fluid occurs—may be linked to AEs like dehydration. Patients with intestinal failure due to low intake issues are at an increased risk of dehydration (Jeppesen et al., 2012). Insufficient fluid intake could increase the supersaturation of salts that form stones in the urine, thereby escalating the risk of kidney stones. Nephrolithiasis are related to calcium excretion in the urine and absorption in the intestines. In the gut, calcium binds to unabsorbed fatty acids, and the remaining free oxalates passively diffuse into the colon and are absorbed before being filtered by the kidney, where oxalates combine with calcium resulting in oxalate Nephrolithiasis (Nightingale et al., 1992). The amount of urinary calcium excreted is affected by the reabsorption of the proximal and distal tubules of the kidney, with the latter’s reabsorption related to active vitamin D. Studies have found a correlation between GLP-2 treatment and increased expression of the vitamin D receptor (VDR) in the ileum. Enhanced active enteral tissue VDR can elevate calcium uptake and reduce the amount of calcium reabsorbed by the kidney tubules (Huang et al., 2023; Li et al., 1993). Additionally, research has also discovered a relationship between VDR polymorphisms and the occurrence of kidney stones, but the specific mechanism of this link remains unclear (Letavernier et al., 2016).

Within the top 30 proportionally highest reported PTs, vascular device infections were prominent (Figure 3). Although not explicitly described in the instruction manuals, numerous vascular device infection events have been reported in relevant literature (Jeppesen et al., 2012; Pape et al., 2020), aligning well with the substantial number observed in our study. Infections may be connected with PN patients underlying disease conditions, total parenteral nutrition patients are prone to such infections. Epidemiological surveys revealed that SBS patients have an incidence rate of venous catheter-related infections of 0.19–11.5 per 1,000 catheter days (Bond et al., 2018; Burden et al., 2018; Deutsch et al., 2020; Dibb et al., 2016; Schwartz et al., 2016). In a safety study of the drug teduglutide in children, 28.1% (36 out of 128) of patients experienced device-related fever (Hill et al., 2021). In another randomized controlled trial study of 173 individuals treated with teduglutide for up to 2.5 years, 24.9% (43 out of 173) had Catheter-related bloodstream infections at their last follow-up (Pape et al., 2020). However, improvement of bowel adaptation and reduction of parenteral support reliance could potentially decrease the risk of vascular device infections complications in SBS patients, particularly if they can achieve enteral autonomic nutrition earlier and allow the removal of parenteral intravenous catheters (O'Keefe et al., 2013). Vascular device infections are associated with a number of factors, such as duration of antibiotic use, degree of dependence on parenteral nutrition, and relapse after discontinuation of medication for reintroduction of parenteral nutrition catheters (Tribler et al., 2018). There is a lack of sufficient studies at this stage to examine whether the use of Teduglutide is associated with the development of catheter-related infections in patients with short bowel syndrome, but the analysis of our pharmacovigilance study revealed a strong signal for vascular device infections AEs, suggesting cautious cognizance of the potential correlation between teduglutide and this adverse event.

The top five high-signal events were mainly associated with stoma output and complications (Table 4). Variations in stoma output are correlated with intestinal motility. Multiple studies have shown that GLP-2 can influence intestinal motor activity, extending the time for liquid phase gastric emptying and solid-phase intestinal and total gastrointestinal emptying (Martin et al., 2006). Furthermore, GLP-2 appears to synergize with glucagon-like peptide-1 (GLP-1), inhibiting small intestinal myoelectric activity in rats (Bozkurt et al., 2002), and *in vitro* experiments found that GLP-2 can inhibit cholinergic contractile movements generated spontaneously or by electrical stimuli in the intestines (Amato et al., 2010). If patients commonly use loperamide or similar drugs that suppress intestinal transport, the cumulative effect of this suppression will reduce stoma output. However, An increase in stoma output might be correlated with patient heterogeneity, and the characteristics of the stoma location in SBS patients might have a certain connection with the response to teduglutide. Specifically, compared to patients who have preserved the terminal ileum and colon, jejunostomy patients usually show accelerated gastric emptying and excessive gastric acid production, resulting in increased output. This may be related to the loss of endogenous hormone secretion functions, as well as neuroendocrine feedback signals from the proximal and distal gastrointestinal tract (Chen et al., 2018). Research has found that patients who take teduglutide have a higher risk of stoma complications than those in the placebo group (37.8% vs13.6%), suggesting that the use of teduglutide might be related to the occurrence of stoma complications (Pape et al., 2020). GLP-2 can can stimulate colonic mucosal growth and improve mesenteric artery blood flow. Following subcutaneous injection of GLP-2, the jejunal mucosa’s microcirculation greatly improves, which appeared to result in reddening and enlargement of the stoma papillae (Hoyerup et al., 2013). In addition, research suggests that teduglutide can visibly increase villous height, crypt depth, and mitotic index, inducing growth of the small intestine mucosa at the terminal end of the jejunostomy patient, which can be observed in the clinical widening of the stoma (Bremholm et al., 2011; Guan et al., 2003).

While no high signals for neoplasms were observed at the SOC level, the identification of polyp-related AEs at the PT level prompts concerns regarding teduglutide’s long-term effects on intestinal epithelial cell proliferation and its potential tumorigenic risk. It is recommended in the instructions that colonoscopy should be performed after 1 year of drug treatment. If intestinal tumors are found, the medication should be discontinued. Concerns concerning the medication for intestine-associated neoplasia are raised by the growth factor GLP-2, which encourages the proliferation of intestinal epithelial cells and may cause enhanced tumor growth in colonic tissues. Research found that IGF-I (insulin like growth factor, IGF) and β-catenin are involved in GLP-2’s induction of intestinal mucosa growth. IGF-I has been proven to be a growth factor, and β-catenin is a known oncogene. It is often overactivated or downregulated in colorectal cancer, possibly leading to an expansion of the epithelial surface area and corresponding morphological changes (Dubé et al., 2008). Teduglutide does not downregulate endogenous GLP-2, and the mucosal proliferation caused by drug treatment might release more GLP-2 (Lambe et al., 2023). Therefore, long-term use of GLP-2 analogs might increase the occurrence of colonic polyp adenomas, and we recommend that during long-term use, follow-ups and close monitoring of AEs related to colonoscopy are necessary.

In this study, there was a higher proportion of serious reports compared to non-serious reports in females, which is similar to the results in Table 1 of a higher number of adverse events in females. Few studies have evaluated the effect of gender on adverse events associated with teduglutide, and further prospective studies are needed to determine whether gender is an important factor in clinical practice. A systematic review of catheter-associated infections in patients receiving home parenteral nutrition identified prolonged treatment time as a risk factor for increased catheter sepsis infections (Dreesen et al., 2013). The majority of adverse events in this study occurred after 1 year, and catheter-related infections were one of the most common adverse events among those with severe reports. Therefore, catheter care for patients is critical and ongoing follow-up is necessary for patients on long-term medication.

Teduglutide is increasingly being used in pediatric patients and therefore differences between children and adults need to be considered when assessing the safety of the drug. We analyzed data on AEs in the pediatric subgroup and found that AEs were more common in pediatric patients for vascular device infection, pyrexia and vomiting, which is in line with findings on the safety of teduglutide drugs in pediatric patients. (C et al., 2021, Kocoshis et al., 2020). The above adverse reactions are more common in children, whereas abnormal pain, nausea and dehydration are more common in adult patients. These differences have not been reported in the literature and may be related to different characteristics at different stages of physical development. In conclusion, our study provides a new basis for monitoring adverse effects in children.

## 5 Limitations

Our investigation encountered several inherent limitations. The FAERS database is a spontaneous reporting system, and 40.35% of the reporters to this study were patients, who are susceptible to bias based on concerns and fears about the relationship between teduglutide and AEs, rather than actual medication-related AEs. It is our contention that the concerns and fears expressed by reporters are frequently related to a lack of knowledge or skill in interpreting signs and symptoms. Consequently, the findings and interpretations presented in this study should be regarded as preliminary and not as definitive evidence. It is important to note that the conclusions reached are based on the limitations of statistical inference with large amounts of data with bias and noise. This may be related to potential errors, sample characteristics, and the limitations of data mining translations in science and practice. In addition, characterising the entirety of the treatment regimen with teduglutide solely in terms of adverse event data, without considering the patient’s underlying condition or concomitant medications, precluded us from conducting a comprehensive analysis of causality. It's also worth noting that many of the symptoms listed as AEs (hypovolaemia, dehydration, weight decreased) are actually the result of mismanagement while taking teduglutide (overly aggressive discontinuation of PN, or failure of the drug itself). Finally, the information in the reports used for the analyses was often not medically substantiated or verified, and we were unable to identify the full target population for teduglutide and could not calculate the incidence of AEs. Based on these factors, there is a high likelihood that our reports are in error and a cautious approach is needed to account for teduglutide-associated AEs. Despite these limitations, our findings underscore the importance of vigilant post-marketing surveillance and the need for extensive prospective studies to elucidate these preliminary observations.

## 6 Conclusion

Leveraging a substantial real-world pharmacovigilance database, our study conducted a thorough evaluation of AEs associated with teduglutide, corroborating known AEs while also unveiling previously unreported ones. Notably, kidney disease emerged as a potential new signal at the SOC level, while at the PTs level, nephrolithiasis represents a novel signals. The essential nature of safety assessments in clinical trials, often limited by selective population cohorts, highlights the potential for discrepancies between reported AEs in clinical settings and real-world experiences. Thus, ongoing post-marketing surveillance is critical, along with comprehensive research to validate our findings, ensuring a complete understanding of teduglutide’s safety profile.

## Data Availability

Publicly available databases were used for all data analyses in this study. The original contributions presented in the study are included in the website: https://fis.fda.gov/extensions/FPD-QDE-FAERS/FPD-QDE-FAERS.html.
