# Supplementary material for: The real-world analysis of adverse events with teduglutide: a pharmacovigilance study based on the FAERS database
Source: Front Pharmacol. 2024 Sep 12;15:1404658. doi: 10.3389/fphar.2024.1404658 (PMC11424547; doi:10.3389/fphar.2024.1404658)
Supplement: Supplementary file 1 [file Table1.pdf]

Table S1 The two-by-two table of disproportionality method.

	"Reports",adverse events of interest	"Non-reports", all other adverse events
Drug investigated	a	b
Other reference drugs	c	d
Total	a+c	b+d

Table S2 Disproportionality methods, formulas, and thresholds.

Algorithms	equation	criteria
ROR	$ROR = \frac{(a/c)}{b/d} = \frac{a*d}{b*c}$ $SE(lnROR) = \sqrt{(\frac{1}{a} + \frac{1}{b} + \frac{1}{c} + \frac{1}{d})}$ $95\%CI = ROR * e^{\pm 1.96\sqrt{\frac{1}{a}+\frac{1}{b}+\frac{1}{c}+\frac{1}{d}}}$	$a \geqslant 3$ and 95%CI(lower bound)>1
PRR	PRR $\frac{a/(a+b)}{c/(c+d)}$ $\mathrm{SE(lnPRR)} = \sqrt{\left(\frac{1}{a} - \frac{1}{a+b} + \frac{1}{c} - \frac{1}{c+d}\right)}$ $95\%CI = PRR * e^{\pm 1.96\sqrt{(\frac{1}{a}-\frac{1}{a+b}+\frac{1}{c}-\frac{1}{c+d})}}$ $x^2 = \frac{(a+b+c+d)(ad-bc)^2}{(a+b)(c+d)(a+c)(b+d)}$	$a \geqslant 3$ and 95%CI(lower bound)>1
BCPN	$IC = \log_2 \frac{p(x,y)}{p(x)p(y)} = \log_2 \frac{a(a+b+c+d)}{(a+b)(a+c)}$ $E(IC) =$	$a \geqslant 3$ and 95% CI (IC025)>0

$$\log_2 \frac{(a+\gamma 11)(a+b+c+d+\alpha)(a+b+c+d+\beta)}{(a+b+c+d+\gamma)(a+b+\alpha 1)(a+c+\beta 1)}$$

$$V(IC) = \frac{1}{(ln2)^2}\left\{\left[\frac{(a+b+c+d)-a+\gamma-\gamma 11}{(a+\gamma 11)(1+a+b+c+d+\gamma)}\right]+\left[\frac{(a+b+c+d)-(a+b)+\alpha-\alpha 1}{(a+b+\alpha 1)(1+a+b+c+d+\alpha)}\right]+\left[\frac{(a+b+c+d)-(a+c)+\beta-\beta 1}{(a+c+\beta 1)(1+a+b+c+d+\beta)}\right]\right\}$$

$$\gamma = \gamma 11 \frac{(a+b+c+d+\alpha)(a+b+c+d+\beta)}{(a+b+\alpha 1)(a+c+\beta 1)} \qquad IC-2SD = E(IC) - 2\sqrt{V(IC)}$$

MGPS	$EBGM = \frac{a(a+b+c+d)}{(a+c)(a+b)}$ $\quad 95\%CI = e^{ln(EBGM)\pm 1.96\sqrt{\frac{1}{a}+\frac{1}{b}+\frac{1}{c}+\frac{1}{d}}}$	$a \geqslant 0$ and EBGM05>2

ROR, reporting odds ratio; PRR, proportional reporting ratio; CI, confidence interval; x^2, chi-square;BCPN, bayesian confidence propagation neural network;IC, information component; IC-2SD, the lower confidence interval of IC; MGPS, multi-item gamma Poisson shrinker; EBGM, empirical Bayesian geometric mean; EBGM05, the lower 95% one-sided CI of EBGM.

Table S3: Signal strength of reports of Teduglutide at the Preferred Term (PT) level.

Soc	PT	Case	ROR (95%CI)	PRR (95%CI)	EBGM (EBGM05)	χ^2	IC (IC025)
Investigations	Weight decreased	805	4.96(4.63-5.32)	4.88(4.55-5.23)	4.86(4.58)	2480.75	4.86(4.58)
Gastrointestinal disorders	Abdominal pain	773	5.93(5.52-6.37)	5.83(5.43-6.26)	5.8(5.46)	3085.52	5.8(5.46)
Infections and infestations	Vascular device infection	683	343.35(314.94-374.33)	336.98(309.1-367.38)	257.96(239.98)	174996.6	257.96(239.98)
Surgical and medical procedures	Hospitalisation	623	6.58(6.07-7.12)	6.48(5.99-7.02)	6.45(6.03)	2878.2	6.45(6.03)
Metabolism and nutrition disorders	Dehydration	596	8.17(7.54-8.87)	8.06(7.43-8.74)	8.01(7.48)	3664.67	8.01(7.48)
Investigations	Weight increased	564	4.45(4.1-4.84)	4.4(4.05-4.78)	4.38(4.09)	1480.09	4.38(4.09)
Gastrointestinal disorders	Abdominal distension	482	8.14(7.43-8.9)	8.04(7.35-8.8)	7.99(7.41)	2955.08	7.99(7.41)

Infections and infestations	Device related infection	448	45.17(41.08-49.68)	44.63(40.59-49.08)	42.93(39.64)	18367.11	42.93(39.64)
Infections and infestations	Sepsis	369	5.82(5.25-6.45)	5.77(5.21-6.39)	5.74(5.27)	1449.61	5.74(5.27)
Gastrointestinal disorders	Intestinal obstruction	301	14.34(12.79-16.07)	14.23(12.69-15.95)	14.06(12.78)	3656.71	14.06(12.78)
Injury, poisoning and procedural complications	Stoma complication	278	664.41(572.36-771.26)	659.38(568.03-765.42)	412.04(363.71)	114099.16	412.04(363.71)
Gastrointestinal disorders	Flatulence	247	7.89(6.96-8.95)	7.85(6.92-8.9)	7.8(7.02)	1466.45	7.8(7.02)
Surgical and medical procedures	Therapy interrupted	210	6.6(5.76-7.56)	6.56(5.73-7.52)	6.53(5.83)	985.51	6.53(5.83)
Infections and infestations	Device related sepsis	196	166.87(143.54-193.98)	165.98(142.78-192.95)	144.28(127.2)	27914.37	144.28(127.2)
Gastrointestinal disorders	Crohn's disease	180	4.66(4.02-5.39)	4.64(4.01-5.37)	4.62(4.09)	512.42	4.62(4.09)

Renal and urinary disorders	Nephrolithiasis	146	5.47(4.65-6.44)	5.45(4.63-6.42)	5.43(4.74)	528.63	5.43(4.74)
General disorders and administration site conditions	Obstruction	135	64.92(54.56-77.25)	64.68(54.36-76.97)	61.14(52.86)	7993.22	61.14(52.86)
Injury, poisoning and procedural complications	Gastrointestinal stoma complication	123	139.13(115.3-167.88)	138.67(114.92-167.32)	123.2(105.28)	14923	123.2(105.28)
Gastrointestinal disorders	Abnormal faeces	112	22.9(18.99-27.62)	22.83(18.93-27.54)	22.39(19.14)	2290.85	22.39(19.14)
General disorders and administration site conditions	Complication associated with device	111	8.11(6.73-9.78)	8.09(6.71-9.75)	8.04(6.87)	685.04	8.04(6.87)
Metabolism and nutrition disorders	Fluid retention	110	3.36(2.79-4.05)	3.35(2.78-4.05)	3.35(2.86)	181.36	3.35(2.86)
Investigations	Gastrointestinal stoma output increased	107	816.62(635.75-1048.96)	814.25(633.9-1045.9)	467.56(379.19)	49861.35	467.56(379.19)

Gastrointestinal disorders	Small intestinal obstruction	106	15.71(12.97-19.04)	15.67(12.93-18.99)	15.46(13.17)	1435.45	15.46(13.17)
Product issues	Product availability issue	105	9.19(7.59-11.14)	9.17(7.57-11.12)	9.1(7.75)	758.23	9.1(7.75)
Metabolism and nutrition disorders	Hypervolaemia	94	7.14(5.83-8.75)	7.13(5.82-8.73)	7.09(5.98)	492.15	7.09(5.98)
Infections and infestations	Staphylococcal infection	92	5.18(4.22-6.35)	5.17(4.21-6.34)	5.15(4.33)	307.73	5.15(4.33)
Metabolism and nutrition disorders	Weight fluctuation	91	13.67(11.11-16.81)	13.64(11.09-16.78)	13.48(11.34)	1052.96	13.48(11.34)
Musculoskeletal and connective tissue disorders	Fistula	90	14.22(11.55-17.51)	14.19(4.36-6.59)	14.02(11.78)	1089.2	14.02(11.78)
Investigations	Blood potassium decreased	90	5.37(4.36-6.61)	5.36(11.52-17.47)	5.34(4.49)	317.7	5.34(4.49)

Metabolism and nutrition disorders	Hypokalaemia	89	3.52(2.86-4.33)	3.51(2.85-4.33)	3.5(2.94)	159.51	3.5(2.94)
Injury, poisoning and procedural complications	Stoma site haemorrhage	88	87.63(70.51-108.91)	87.43(70.35-108.65)	81.04(67.56)	6963.2	81.04(67.56)
Hepatobiliary disorders	Cholelithiasis	88	5.65(4.58-6.96)	5.64(4.57-6.95)	5.61(4.71)	333.98	5.61(4.71)
Social circumstances	Insurance issue	86	14.34(11.59-17.75)	14.31(11.57-17.71)	14.14(11.83)	1051.22	14.14(11.83)
Gastrointestinal disorders	Frequent bowel movements	83	5.3(4.27-6.58)	5.29(4.27-6.57)	5.27(4.4)	287.79	5.27(4.4)
Product issues	Needle issue	83	4.8(3.87-5.96)	4.79(3.86-5.95)	4.78(3.99)	248.25	4.78(3.99)
Metabolism and nutrition disorders	Malnutrition	79	13.79(11.05-17.23)	13.77(11.02-17.19)	13.61(11.3)	923.9	13.61(11.3)
Investigations	Blood magnesium decreased	78	15.49(12.38-19.37)	15.46(12.36-19.33)	15.26(12.65)	1040.32	15.26(12.65)

Metabolism and nutrition disorders	Electrolyte imbalance	66	10.56(8.28-13.46)	10.54(8.27-13.44)	10.45(8.53)	564.65	10.45(8.53)
Infections and infestations	Bacteraemia	64	9.77(7.64-12.5)	9.76(4.26-6.97)	9.68(7.88)	498.56	9.68(7.88)
General disorders and administration site conditions	Hernia	64	5.46(4.27-6.98)	5.45(7.63-12.48)	5.43(4.42)	231.47	5.43(4.42)
Product issues	Product distribution issue	61	18.94(14.7-24.4)	18.91(14.68-24.36)	18.61(15.05)	1017.23	18.61(15.05)
Hepatobiliary disorders	Gallbladder disorder	53	6.42(4.9-8.41)	6.41(4.49-7.71)	6.38(5.09)	240.74	6.38(5.09)
Injury, poisoning and procedural complications	Post procedural complication	53	5.89(4.5-7.72)	5.89(4.89-8.4)	5.86(4.68)	213.93	5.86(4.68)
Hepatobiliary disorders	Cholecystitis	52	8.98(6.83-11.8)	8.97(6.82-11.78)	8.9(7.08)	365.06	8.9(7.08)
Investigations	Urine output decreased	50	10.95(8.29-14.47)	10.94(8.28-14.45)	10.84(8.58)	447.09	10.84(8.58)

Investigations	Lipase increased	49	12.38(9.34-16.4)	12.36(9.33-16.38)	12.23(9.66)	505.98	12.23(9.66)
Gastrointestinal disorders	Faeces hard	48	21.68(16.29-28.86)	21.65(7.1-12.54)	21.25(16.73)	927.37	21.25(16.73)
Gastrointestinal disorders	Large intestine polyp	48	9.45(7.11-12.56)	9.44(16.27-28.82)	9.37(7.38)	359.15	9.37(7.38)
Social circumstances	Inability to afford medication	47	8.83(6.62-11.76)	8.82(6.62-11.75)	8.75(6.88)	323.13	8.75(6.88)
Metabolism and nutrition disorders	Appetite disorder	46	11.67(8.72-15.6)	11.65(8.71-15.59)	11.54(9.05)	443.4	11.54(9.05)
Metabolism and nutrition disorders	Increased appetite	45	4.78(3.57-6.41)	4.78(3.56-6.4)	4.76(3.73)	133.85	4.76(3.73)
Investigations	Gastrointestinal stoma output decreased	44	1027.09(680.76-1549.62)	1025.86(679.94-1547.77)	530.33(375.91)	23267.66	530.33(375.91)
Gastrointestinal disorders	Ileus	44	7.09(5.27-9.54)	7.08(5.27-9.53)	7.05(5.5)	228.48	7.05(5.5)

Infections and infestations	Bacterial infection	44	4.36(3.24-5.86)	4.36(3.24-5.86)	4.34(3.39)	113.39	4.34(3.39)
Product issues	Syringe issue	44	4.1(3.05-5.51)	4.09(3.04-5.5)	4.08(3.19)	102.5	4.08(3.19)
Psychiatric disorders	Eating disorder	42	3.22(2.38-4.36)	3.21(2.37-4.35)	3.21(2.49)	63.9	3.21(2.49)
Investigations	Urine output increased	41	25.7(18.85-35.04)	25.67(6.36-11.76)	25.11(19.37)	949.99	25.11(19.37)
General disorders and administration site conditions	Polyp	41	10.32(7.58-14.03)	10.3(7.58-14.02)	10.22(7.9)	341.3	10.22(7.9)
Gastrointestinal disorders	Bowel movement irregularity	41	8.66(6.37-11.78)	8.65(18.83-35)	8.59(6.64)	275.25	8.59(6.64)
Investigations	Blood iron decreased	40	5.63(4.13-7.69)	5.63(2.4-4.47)	5.6(4.32)	151.49	5.6(4.32)
Infections and infestations	Kidney infection	40	3.28(2.4-4.47)	3.28(4.12-7.68)	3.27(2.52)	63.15	3.27(2.52)

Injury, poisoning and procedural complications	Stoma site pain	38	36.63(26.51-50.61)	36.59(2.56-4.84)	35.44(27.04)	1273.03	35.44(27.04)
Infections and infestations	Gastroenteritis viral	38	3.52(2.56-4.85)	3.52(26.48-50.56)	3.51(2.69)	68.43	3.51(2.69)
Infections and infestations	Device related bacteraemia	37	279.9(195.06-401.65)	279.62(2.16-4.12)	222.98(164.83)	8183.84	222.98(164.83)
Infections and infestations	Catheter site infection	37	15.71(11.36-21.74)	15.7(11.34-21.72)	15.49(11.8)	501.98	15.49(11.8)
Investigations	Gastrointestinal stoma output abnormal	34	867.26(553.03-1360.03)	866.45(552.52-1358.77)	484.31(332.37)	16413.45	484.31(332.37)
Infections and infestations	Gastroenteritis	34	4.26(3.04-5.97)	4.26(3.04-5.97)	4.25(3.2)	84.51	4.25(3.2)
Gastrointestinal disorders	Short-bowel syndrome	33	87.42(61.32-124.64)	87.35(3.59-7.11)	80.97(60.18)	2608.91	80.97(60.18)
Gastrointestinal disorders	Gastrointestinal pain	33	5.05(3.59-7.11)	5.05(61.27-124.53)	5.03(3.78)	106.64	5.03(3.78)

Injury, poisoning and procedural complications	Stoma obstruction	32	265.88(180.68-391.27)	265.65(180.52-390.93)	214.01(154.9)	6790.74	214.01(154.9)
Gastrointestinal disorders	Malabsorption	32	9.66(6.82-13.68)	9.65(6.81-13.67)	9.57(7.15)	245.95	9.57(7.15)
Infections and infestations	Gastrointestinal bacterial overgrowth	31	58.62(40.84-84.14)	58.57(40.8-84.07)	55.65(41.12)	1665.2	55.65(41.12)
General disorders and administration site conditions	Device related thrombosis	31	49.92(34.83-71.56)	49.88(2.68-5.42)	47.75(35.33)	1420.35	47.75(35.33)
Product issues	Device occlusion	31	3.81(2.68-5.42)	3.81(34.8-71.51)	3.8(2.83)	63.98	3.8(2.83)
Gastrointestinal disorders	Enterocutaneous fistula	30	64.89(44.89-93.8)	64.84(44.86-93.72)	61.27(45.02)	1780.36	61.27(45.02)
Investigations	Blood potassium increased	29	3.31(2.3-4.76)	3.31(1.92-3.97)	3.3(2.43)	46.52	3.3(2.43)
Investigations	Faecal volume increased	28	100.35(68.14-147.78)	100.27(68.08-147.67)	91.95(66.51)	2521.17	91.95(66.51)

Infections and infestations	Abdominal abscess	28	11.42(7.87-16.57)	11.41(7.86-16.56)	11.3(8.28)	263.22	11.3(8.28)
Gastrointestinal disorders	Duodenal polyp	27	266.74(175.14-406.26)	266.55(2.85-6.06)	214.59(150.92)	5745.4	214.59(150.92)
Infections and infestations	Overgrowth bacterial	27	226.02(149.34-342.07)	225.85(149.23-341.81)	187.43(132.51)	5011.33	187.43(132.51)
Metabolism and nutrition disorders	Weight gain poor	27	21.13(14.44-30.93)	21.12(175.01-405.96)	20.74(15.08)	507.71	20.74(15.08)
Investigations	Blood calcium decreased	27	4.15(2.85-6.06)	4.15(14.43-30.91)	4.14(3.02)	64.39	4.14(3.02)
Infections and infestations	Haematological infection	26	51.37(34.66-76.13)	51.34(34.64-76.08)	49.08(35.32)	1225.81	49.08(35.32)
Surgical and medical procedures	Central venous catheterisation	24	11.96(8-17.89)	11.95(5.73-12.8)	11.84(8.45)	238.32	11.84(8.45)
Infections and infestations	Endocarditis	24	8.57(5.74-12.81)	8.57(2.99-6.67)	8.51(6.08)	159.17	8.51(6.08)

Infections and infestations	Gastrointestinal infection	24	4.47(2.99-6.67)	4.46(7.99-17.88)	4.45(3.18)	64.25	4.45(3.18)
Injury, poisoning and procedural complications	Stoma site oedema	23	240.18(152.94-377.19)	240.03(5.44-12.36)	197.08(135.09)	4491.13	197.08(135.09)
Gastrointestinal disorders	Pancreatic disorder	23	8.65(5.74-13.05)	8.65(4.83-10.96)	8.59(6.09)	154.38	8.59(6.09)
Gastrointestinal disorders	Abdominal hernia	23	8.2(5.44-12.37)	8.2(5.74-13.04)	8.15(5.78)	144.31	8.15(5.78)
Gastrointestinal disorders	Gastrointestinal motility disorder	23	7.28(4.83-10.97)	7.27(152.85-376.96)	7.23(5.13)	123.65	7.23(5.13)
Injury, poisoning and procedural complications	Stoma site inflammation	22	41.52(27.12-63.56)	41.49(5.85-13.54)	40.02(28.02)	837.68	40.02(28.02)
Gastrointestinal disorders	Gastric polyps	22	17.07(11.2-26.01)	17.06(5.88-13.61)	16.82(11.82)	327.55	16.82(11.82)
Investigations	Amylase increased	22	8.95(5.88-13.61)	8.94(27.1-63.52)	8.88(6.25)	153.94	8.88(6.25)

Infections and infestations	Staphylococcal sepsis	22	8.9(5.85-13.55)	8.9(11.2-26)	8.84(6.22)	153.02	8.84(6.22)
General disorders and administration site conditions	Hunger	22	3.59(2.36-5.45)	3.59(2.36-5.45)	3.58(2.52)	40.91	3.58(2.52)
Infections and infestations	Staphylococcal bacteraemia	21	9.44(6.15-14.51)	9.44(3.89-9.17)	9.37(6.54)	157.1	9.37(6.54)
Investigations	Blood albumin decreased	21	5.98(3.89-9.18)	5.97(6.14-14.51)	5.95(4.15)	86.51	5.95(4.15)
Product issues	Product container issue	21	3.75(2.44-5.75)	3.75(2.17-5.12)	3.74(2.61)	42.16	3.74(2.61)
Gastrointestinal disorders	Intestinal perforation	21	3.33(2.17-5.12)	3.33(2.44-5.75)	3.33(2.32)	34.2	3.33(2.32)
Injury, poisoning and procedural complications	Stoma prolapse	19	1096.38(580.43-2070.94)	1095.81(2.14-5.26)	548.4(322.1)	10391.2	548.4(322.1)
Investigations	Faecal volume	19	86.08(53.96-137.32)	86.03(4.65-11.46)	79.84(54.01)	1480.64	79.84(54.01)

	decreased						
Metabolism and nutrition disorders	Iron deficiency	19	7.3(4.65-11.46)	7.3(53.93-137.25)	7.25(4.97)	102.54	7.25(4.97)
Surgical and medical procedures	Cholecystectomy	19	3.35(2.14-5.26)	3.35(580.13-2069.86)	3.35(2.29)	31.28	3.35(2.29)
Gastrointestinal disorders	Gastrointestinal obstruction	18	12.28(7.72-19.54)	12.27(4.18-10.56)	12.15(8.24)	184.34	12.15(8.24)
Infections and infestations	Cholecystitis infective	18	11.42(7.18-18.17)	11.41(7.71-19.53)	11.31(7.67)	169.29	11.31(7.67)
Hepatobiliary disorders	Bile duct stone	18	10.72(6.74-17.05)	10.71(2.43-6.12)	10.62(7.2)	157.01	10.62(7.2)
Gastrointestinal disorders	Gastrointestinal sounds abnormal	18	6.65(4.18-10.56)	6.64(2.73-6.89)	6.61(4.48)	85.78	6.61(4.48)
Hepatobiliary disorders	Cholecystitis acute	18	6.38(4.01-10.14)	6.38(4.01-10.14)	6.35(4.31)	81.18	6.35(4.31)

Renal and urinary disorders	Hydronephrosis	18	4.34(2.73-6.89)	4.34(6.73-17.05)	4.32(2.93)	46.02	4.32(2.93)
Immune system disorders	Multiple allergies	18	3.86(2.43-6.12)	3.85(7.17-18.16)	3.84(2.61)	37.91	3.84(2.61)
Infections and infestations	Rhinovirus infection	17	8.07(5.01-13.01)	8.07(3.64-9.43)	8.02(5.38)	104.5	8.02(5.38)
Gastrointestinal disorders	Intestinal stenosis	17	6.5(4.04-10.48)	6.5(2.67-6.92)	6.47(4.34)	78.65	6.47(4.34)
Gastrointestinal disorders	Faecaloma	17	5.86(3.64-9.44)	5.86(4.03-10.47)	5.83(3.91)	68.1	5.83(3.91)
Metabolism and nutrition disorders	Hypoalbuminaemia	17	4.3(2.67-6.92)	4.3(2.18-5.66)	4.29(2.88)	42.86	4.29(2.88)
Gastrointestinal disorders	Gastrointestinal inflammation	17	3.51(2.18-5.66)	3.51(1.87-4.85)	3.51(2.35)	30.47	3.51(2.35)
General disorders and administration site conditions	Catheter site inflammation	16	42.68(25.9-70.33)	42.66(2.47-6.61)	41.1(27.06)	626.54	41.1(27.06)

Vascular disorders	Jugular vein thrombosis	16	16.78(10.24-27.5)	16.78(5.02-13.42)	16.54(10.94)	233.83	16.54(10.94)
Gastrointestinal disorders	Subileus	16	13.97(8.53-22.87)	13.96(2.15-5.74)	13.8(9.13)	190.08	13.8(9.13)
Investigations	Weight abnormal	16	10.32(6.31-16.88)	10.31(25.89-70.3)	10.23(6.77)	133.32	10.23(6.77)
Gastrointestinal disorders	Abdominal adhesions	16	8.21(5.02-13.43)	8.21(10.24-27.49)	8.15(5.4)	100.53	8.15(5.4)
Metabolism and nutrition disorders	Acidosis	16	4.04(2.48-6.61)	4.04(8.52-22.86)	4.03(2.67)	36.52	4.03(2.67)
Gastrointestinal disorders	Impaired gastric emptying	16	3.51(2.15-5.74)	3.51(6.3-16.88)	3.5(2.32)	28.64	3.5(2.32)
Gastrointestinal disorders	Dumping syndrome	15	87.93(51.96-148.81)	87.9(5.69-15.73)	81.45(52.44)	1192.97	81.45(52.44)
Gastrointestinal disorders	Gastrointestinal fistula	15	26.78(16.04-44.7)	26.77(6.46-17.85)	26.16(17.04)	363.25	26.16(17.04)
Gastrointestinal disorders	Rectal polyp	15	24.32(14.58-40.58)	24.32(2.39-6.58)	23.81(15.52)	328.07	23.81(15.52)

Injury, poisoning and procedural complications	Stoma site erythema	15	10.74(6.46-17.86)	10.74(51.94-148.75)	10.64(6.95)	131.16	10.64(6.95)
Investigations	Blood potassium abnormal	15	9.47(5.69-15.74)	9.46(16.04-44.68)	9.39(6.14)	112.56	9.39(6.14)
Metabolism and nutrition disorders	Hyperphagia	15	7.53(4.53-12.52)	7.53(4.53-12.51)	7.49(4.89)	84.36	7.49(4.89)
Infections and infestations	Klebsiella infection	15	5.55(3.34-9.21)	5.55(14.58-40.56)	5.52(3.61)	55.61	5.52(3.61)
Gastrointestinal disorders	Proctalgia	15	3.96(2.39-6.58)	3.96(3.34-9.21)	3.95(2.59)	33.11	3.95(2.59)
Infections and infestations	Stoma site infection	14	15.35(9.06-26.01)	15.34(8.1-23.25)	15.14(9.74)	185.1	15.14(9.74)
Gastrointestinal disorders	Pancreatic cyst	14	13.73(8.1-23.26)	13.72(1.86-5.31)	13.56(8.73)	163.09	13.56(8.73)
Injury, poisoning and procedural complications	Stoma site discharge	14	6.66(3.94-11.26)	6.66(3.94-11.26)	6.62(4.27)	66.88	6.62(4.27)

Gastrointestinal disorders	Intestinal ischaemia	14	4.82(2.85-8.15)	4.82(2.85-8.14)	4.8(3.09)	42.16	4.8(3.09)
Infections and infestations	Respiratory syncytial virus infection	14	3.26(1.93-5.51)	3.26(9.05-26)	3.25(2.1)	21.84	3.25(2.1)
Injury, poisoning and procedural complications	Stoma site discomfort	13	83.83(47.69-147.36)	83.8(7.26-21.68)	77.92(48.6)	987.97	77.92(48.6)
Infections and infestations	Spinal cord infection	13	31.05(17.89-53.88)	31.04(17.88-53.87)	30.21(19.04)	367.48	30.21(19.04)
Metabolism and nutrition disorders	Fluid imbalance	13	28.05(16.17-48.65)	28.04(4.83-14.4)	27.37(17.26)	330.56	27.37(17.26)
Gastrointestinal disorders	Intestinal polyp	13	12.76(7.38-22.04)	12.75(7.38-22.04)	12.62(7.98)	139.2	12.62(7.98)
Infections and infestations	Intervertebral discitis	13	12.56(7.27-21.69)	12.55(47.67-147.3)	12.42(7.86)	136.64	12.42(7.86)
General disorders and administration	Catheter site pain	13	8.34(4.83-14.4)	8.34(16.17-48.63)	8.29(5.25)	83.35	8.29(5.25)

site conditions							
Gastrointestinal disorders	Intestinal haemorrhage	13	3.89(2.26-6.71)	3.89(2.26-6.71)	3.88(2.46)	27.82	3.88(2.46)
Investigations	Blood magnesium abnormal	12	37.58(21.14-66.82)	37.57(21.13-66.8)	36.36(22.46)	413.01	36.36(22.46)
Injury, poisoning and procedural complications	Stoma site extravasation	12	35.08(19.74-62.33)	35.07(4.77-14.85)	34.01(21.02)	384.82	34.01(21.02)
Product issues	Product reconstitution quality issue	12	17.63(9.97-31.19)	17.63(1.87-5.8)	17.36(10.77)	185.23	17.36(10.77)
Musculoskeletal and connective tissue disorders	Fistula discharge	12	15.87(8.97-28.06)	15.86(9.96-31.18)	15.65(9.71)	164.72	15.65(9.71)
Renal and urinary disorders	Renal colic	12	9.71(5.5-17.14)	9.7(19.73-62.31)	9.63(5.98)	92.87	9.63(5.98)

Gastrointestinal disorders	Infrequent bowel movements	12	8.42(4.77-14.85)	8.41(8.97-28.05)	8.36(5.2)	77.79	8.36(5.2)
Investigations	Blood creatinine abnormal	12	5.77(3.27-10.18)	5.77(5.5-17.13)	5.75(3.58)	47.1	5.75(3.58)
Reproductive system and breast disorders	Breast mass	12	3.29(1.87-5.8)	3.29(3.27-10.18)	3.28(2.04)	19.05	3.28(2.04)
General disorders and administration site conditions	Vascular device occlusion	11	70.51(38.32-129.74)	70.49(23.9-79.79)	66.29(39.8)	708.01	66.29(39.8)
Surgical and medical procedures	Intestinal anastomosis	11	43.69(23.91-79.82)	43.67(7.25-23.8)	42.04(25.39)	441.08	42.04(25.39)
Infections and infestations	Klebsiella bacteraemia	11	22.97(12.64-41.73)	22.96(2.75-8.98)	22.51(13.66)	226.3	22.51(13.66)
Infections and infestations	Fungaemia	11	13.13(7.25-23.8)	13.13(3.65-11.96)	12.99(7.9)	121.82	12.99(7.9)
General disorders and	Physical deconditionin	11	9.55(5.28-17.3)	9.55(2.05-6.7)	9.48(5.77)	83.49	9.48(5.77)

administration site conditions	g						
Investigations	Vitamin b12 decreased	11	6.61(3.65-11.96)	6.61(12.64-41.72)	6.57(4)	52.05	6.57(4)
Investigations	Blood creatine increased	11	4.97(2.75-8.98)	4.96(38.31-129.7)	4.95(3.01)	34.67	4.95(3.01)
Product issues	Product leakage	11	3.71(2.05-6.71)	3.71(5.28-17.29)	3.7(2.25)	21.69	3.7(2.25)
Metabolism and nutrition disorders	Vitamin a deficiency	10	249.12(125.36-495.03)	249.05(3.99-13.83)	203.11(114.34)	2013.02	203.11(114.34)
Surgical and medical procedures	Parenteral nutrition	10	117.86(61.38-226.3)	117.83(5.73-19.91)	106.49(61.69)	1045.91	106.49(61.69)
Hepatobiliary disorders	Biliary dilatation	10	13.87(7.44-25.89)	13.87(6.78-23.6)	13.71(8.14)	117.94	13.71(8.14)
Neoplasms benign, malignant and unspecified (incl cysts and	Colorectal adenoma	10	13.35(7.16-24.91)	13.35(3.31-11.47)	13.2(7.83)	112.85	13.2(7.83)

polyps)							
Metabolism and nutrition disorders	Hypovitaminosis	10	12.66(6.79-23.61)	12.65(7.43-25.88)	12.52(7.43)	106.1	12.52(7.43)
Gastrointestinal disorders	Gastrointestinal wall thickening	10	10.68(5.73-19.92)	10.68(61.37-226.24)	10.59(6.29)	86.9	10.59(6.29)
Gastrointestinal disorders	Volvulus	10	7.43(3.99-13.83)	7.42(7.15-24.9)	7.38(4.39)	55.22	7.38(4.39)
Hepatobiliary disorders	Biliary colic	10	6.16(3.31-11.47)	6.16(125.33-494.9)	6.13(3.64)	42.98	6.13(3.64)
Vascular disorders	Venous thrombosis	10	4.57(2.45-8.5)	4.57(2.34-8.09)	4.55(2.71)	27.75	4.55(2.71)
General disorders and administration site conditions	Systemic inflammatory response syndrome	10	4.35(2.34-8.09)	4.35(2.45-8.5)	4.33(2.58)	25.67	4.33(2.58)
Injury, poisoning and procedural complications	Gastrostomy tube site complication	9	73.62(37.48-144.59)	73.6(2.09-7.76)	69.03(39.24)	603.95	69.03(39.24)

Injury, poisoning and procedural complications	Anastomotic ulcer	9	41.45(21.3-80.65)	41.44(37.47-144.55)	39.96(22.9)	342.22	39.96(22.9)
Gastrointestinal disorders	Faecal vomiting	9	30.26(15.6-58.68)	30.25(11.13-41.65)	29.47(16.93)	247.73	29.47(16.93)
Injury, poisoning and procedural complications	Stoma site reaction	9	21.54(11.13-41.66)	21.53(15.6-58.67)	21.14(12.17)	172.82	21.14(12.17)
Gastrointestinal disorders	Intestinal pseudo-obstruction	9	13.8(7.15-26.63)	13.79(2.74-10.14)	13.63(7.87)	105.47	13.63(7.87)
Vascular disorders	Vena cava thrombosis	9	10.21(5.3-19.69)	10.21(3.5-13)	10.12(5.85)	74.08	10.12(5.85)
Gastrointestinal disorders	Rectal discharge	9	6.75(3.51-13)	6.75(2.64-9.77)	6.72(3.88)	43.82	6.72(3.88)
General disorders and administration site conditions	Catheter site erythema	9	6.63(3.44-12.77)	6.63(5.3-19.68)	6.59(3.81)	42.75	6.59(3.81)

Investigations	Respiratory rate decreased	9	5.63(2.93-10.85)	5.63(3.44-12.76)	5.61(3.24)	34.12	5.61(3.24)
Hepatobiliary disorders	Biliary obstruction	9	5.27(2.74-10.14)	5.27(7.15-26.62)	5.25(3.03)	30.95	5.25(3.03)
General disorders and administration site conditions	Granuloma	9	5.08(2.64-9.77)	5.08(21.3-80.63)	5.06(2.92)	29.32	5.06(2.92)
Metabolism and nutrition disorders	Food intolerance	9	4.03(2.09-7.76)	4.03(2.93-10.84)	4.02(2.32)	20.43	4.02(2.32)
Gastrointestinal disorders	Gastric mucosal hypertrophy	8	63.54(31.15-129.61)	63.53(5.68-22.88)	60.1(33.1)	465.35	60.1(33.1)
Metabolism and nutrition disorders	Protein deficiency	8	37.15(18.36-75.17)	37.15(2.47-9.9)	35.96(19.94)	272.16	35.96(19.94)
Injury, poisoning and procedural complications	Stomal hernia	8	35.64(17.63-72.08)	35.64(3.66-14.7)	34.55(19.16)	260.83	34.55(19.16)
Gastrointestinal disorders	Intestinal prolapse	8	32.36(16.02-65.36)	32.35(31.14-129.58)	31.45(17.46)	236.07	31.45(17.46)

Infections and infestations	Rotavirus infection	8	26.17(12.98-52.77)	26.17(16.01-65.35)	25.58(14.23)	189.14	25.58(14.23)
Investigations	Nutritional condition abnormal	8	21.81(10.83-43.92)	21.81(2.24-8.98)	21.4(11.92)	155.73	21.4(11.92)
Gastrointestinal disorders	Small intestinal stenosis	8	13.23(6.59-26.56)	13.22(2.17-8.7)	13.08(7.3)	89.31	13.08(7.3)
Gastrointestinal disorders	Intestinal dilatation	8	12.16(6.06-24.41)	12.16(6.06-24.41)	12.04(6.72)	81.03	12.04(6.72)
Investigations	Pancreatic enzymes increased	8	11.4(5.68-22.88)	11.4(2.77-11.12)	11.29(6.3)	75.12	11.29(6.3)
Surgical and medical procedures	Intestinal operation	8	9.98(4.97-20.01)	9.97(18.36-75.15)	9.89(5.52)	64.01	9.89(5.52)
Renal and urinary disorders	Ureterolithiasis	8	8.02(4-16.08)	8.02(3.73-14.98)	7.97(4.45)	48.81	7.97(4.45)
Gastrointestinal disorders	Gastric dilatation	8	7.47(3.73-14.98)	7.47(3.13-12.57)	7.43(4.15)	44.56	7.43(4.15)

Surgical and medical procedures	Colostomy	8	7.34(3.66-14.71)	7.34(4-16.08)	7.29(4.08)	43.49	7.29(4.08)
Surgical and medical procedures	Catheter placement	8	6.71(3.35-13.44)	6.71(6.58-26.55)	6.67(3.73)	38.62	6.67(3.73)
Surgical and medical procedures	Ileostomy	8	6.28(3.13-12.58)	6.28(10.83-43.91)	6.25(3.49)	35.28	6.25(3.49)
Gastrointestinal disorders	Large intestinal obstruction	8	5.55(2.77-11.12)	5.55(17.62-72.07)	5.53(3.09)	29.68	5.53(3.09)
Hepatobiliary disorders	Portal hypertension	8	4.95(2.47-9.9)	4.94(2.29-9.18)	4.93(2.76)	25.06	4.93(2.76)
Infections and infestations	Adenovirus infection	8	4.85(2.42-9.71)	4.85(1.82-7.31)	4.83(2.7)	24.31	4.83(2.7)
Gastrointestinal disorders	Varices oesophageal	8	4.81(2.4-9.63)	4.81(3.35-13.44)	4.79(2.68)	24.01	4.79(2.68)
General disorders and administration site conditions	Injection site scar	8	4.59(2.29-9.18)	4.58(12.98-52.76)	4.57(2.56)	22.33	4.57(2.56)

Investigations	Blood creatinine decreased	8	4.48(2.24-8.98)	4.48(2.4-9.63)	4.47(2.5)	21.57	4.47(2.5)
Hepatobiliary disorders	Cholecystitis chronic	8	4.34(2.17-8.7)	4.34(4.97-20.01)	4.33(2.42)	20.51	4.33(2.42)
Gastrointestinal disorders	Dyschezia	8	3.65(1.82-7.31)	3.65(2.42-9.71)	3.64(2.04)	15.34	3.64(2.04)
Injury, poisoning and procedural complications	Parenteral nutrition associated liver disease	7	109.6(50.39-238.39)	109.58(7.59-33.78)	99.71(52.04)	684.67	99.71(52.04)
Surgical and medical procedures	Stoma closure	7	54.8(25.65-117.08)	54.79(4.2-18.57)	52.23(27.67)	352.06	52.23(27.67)
General disorders and administration site conditions	Catheter site thrombosis	7	45.67(21.44-97.27)	45.66(2.02-8.91)	43.87(23.3)	293.53	43.87(23.3)
Infections and infestations	Stoma site abscess	7	41.92(19.71-89.19)	41.92(5.73-25.44)	40.41(21.49)	269.28	40.41(21.49)
Investigations	Body height increased	7	33.36(15.72-70.76)	33.35(9.13-40.69)	32.4(17.27)	213.18	32.4(17.27)

Metabolism and nutrition disorders	Magnesium deficiency	7	25.32(11.97-53.57)	25.32(3.21-14.21)	24.77(13.23)	159.8	24.77(13.23)
Infections and infestations	Infected fistula	7	19.28(9.13-40.7)	19.27(6.79-30.17)	18.96(10.14)	119.18	18.96(10.14)
Infections and infestations	Candida sepsis	7	16.02(7.59-33.78)	16.01(11.97-53.56)	15.8(8.46)	97.12	15.8(8.46)
Neoplasms benign, malignant and unspecified (incl cysts and polyps)	Adenoma benign	7	14.31(6.79-30.17)	14.31(2.11-9.32)	14.14(7.58)	85.55	14.14(7.58)
General disorders and administration site conditions	Catheter site swelling	7	12.08(5.74-25.45)	12.08(21.44-97.26)	11.96(6.41)	70.36	11.96(6.41)
Vascular disorders	Subclavian vein thrombosis	7	11.5(5.46-24.22)	11.5(5.36-23.75)	11.39(6.11)	66.41	11.39(6.11)
Hepatobiliary disorders	Bile duct stenosis	7	11.28(5.36-23.76)	11.28(5.03-22.31)	11.18(5.99)	64.92	11.18(5.99)

Surgical and medical procedures	Covid-19 immunisation	7	10.6(5.03-22.31)	10.59(4.22-18.68)	10.5(5.63)	60.24	10.5(5.63)
Investigations	Blood electrolytes decreased	7	8.88(4.22-18.68)	8.88(5.46-24.22)	8.81(4.73)	48.54	8.81(4.73)
General disorders and administration site conditions	Adhesion	7	8.83(4.2-18.58)	8.83(15.72-70.75)	8.76(4.7)	48.19	8.76(4.7)
Gastrointestinal disorders	Gastrointestinal oedema	7	6.76(3.21-14.21)	6.76(19.7-89.17)	6.72(3.61)	34.13	6.72(3.61)
Gastrointestinal disorders	Duodenitis	7	4.43(2.11-9.32)	4.43(25.64-117.06)	4.42(2.37)	18.54	4.42(2.37)
Gastrointestinal disorders	Pneumatosis intestinalis	7	4.24(2.02-8.91)	4.24(50.38-238.34)	4.23(2.27)	17.27	4.23(2.27)
Gastrointestinal disorders	Small intestine polyp	6	126.46(54.31-294.44)	126.44(6.86-34.39)	113.46(55.94)	669.44	113.46(55.94)
Gastrointestinal disorders	Gastrointestinal hypermotility	6	26.84(11.94-60.33)	26.84(5.74-28.72)	26.22(13.31)	145.67	26.22(13.31)

Surgical and medical procedures	Catheter removal	6	24.36(10.84-54.7)	24.35(4.85-24.21)	23.84(12.12)	131.43	23.84(12.12)
General disorders and administration site conditions	Catheter site related reaction	6	17.58(7.85-39.39)	17.58(7.85-39.38)	17.32(8.82)	92.34	17.32(8.82)
Injury, poisoning and procedural complications	Stoma site irritation	6	15.36(6.86-34.39)	15.36(3.05-15.19)	15.16(7.73)	79.45	15.16(7.73)
Surgical and medical procedures	Gastrointestinal surgery	6	13.48(6.02-30.14)	13.47(3.22-16.02)	13.32(6.79)	68.44	13.32(6.79)
Skin and subcutaneous tissue disorders	Excessive granulation tissue	6	12.84(5.74-28.72)	12.84(11.94-60.32)	12.7(6.48)	64.76	12.7(6.48)
Investigations	Blood magnesium increased	6	12.62(5.64-28.23)	12.62(5.64-28.22)	12.49(6.37)	63.46	12.49(6.37)
Hepatobiliary disorders	Gallbladder enlargement	6	10.83(4.85-24.21)	10.83(2.15-10.67)	10.74(5.48)	53.02	10.74(5.48)

Gastrointestinal disorders	Intestinal fistula	6	7.18(3.22-16.02)	7.18(54.31-294.39)	7.14(3.65)	31.7	7.14(3.65)
Renal and urinary disorders	Calculus urinary	6	6.81(3.05-15.19)	6.81(2.21-11.01)	6.77(3.46)	29.54	6.77(3.46)
Gastrointestinal disorders	Pancreatic failure	6	6.42(2.88-14.31)	6.41(10.84-54.69)	6.38(3.26)	27.26	6.38(3.26)
General disorders and administration site conditions	Infusion site bruising	6	4.94(2.21-11.01)	4.94(2.87-14.31)	4.92(2.51)	18.75	4.92(2.51)
Metabolism and nutrition disorders	Underweight	6	4.79(2.15-10.67)	4.79(6.02-30.14)	4.77(2.44)	17.89	4.77(2.44)
Infections and infestations	Respiratory tract infection viral	6	4.64(2.08-10.36)	4.64(2.08-10.35)	4.63(2.37)	17.08	4.63(2.37)
Surgical and medical procedures	Ileostomy closure	5	33.21(13.64-80.85)	33.21(4.9-28.57)	32.26(15.32)	151.59	32.26(15.32)
Neoplasms benign, malignant and	Gastrointestinal tract adenoma	5	25.97(10.7-63.05)	25.97(2.23-12.92)	25.39(12.09)	117.25	25.39(12.09)

unspecified (incl cysts and polyps)							
Injury, poisoning and procedural complications	Gastrointestinal anastomotic leak	5	22.37(9.23-54.22)	22.36(2.4-13.93)	21.94(10.46)	100	21.94(10.46)
Injury, poisoning and procedural complications	Dialysis related complication	5	20.15(8.32-48.8)	20.14(2.24-13)	19.8(9.44)	89.32	19.8(9.44)
Metabolism and nutrition disorders	Feeding intolerance	5	16.46(6.8-39.8)	16.45(5.86-34.21)	16.22(7.75)	71.5	16.22(7.75)
Infections and infestations	Norovirus infection	5	14.69(6.08-35.51)	14.69(13.64-80.84)	14.51(6.93)	62.94	14.51(6.93)
Investigations	Blood urea nitrogen/creatinine ratio increased	5	14.16(5.86-34.21)	14.16(1.89-10.94)	13.99(6.69)	60.36	13.99(6.69)
Gastrointestinal disorders	Gastric mucosal lesion	5	12.83(5.31-30.99)	12.83(3.31-19.22)	12.69(6.07)	53.92	12.69(6.07)

Infections and infestations	Septic embolus	5	11.84(4.9-28.57)	11.83(2.14-12.39)	11.72(5.61)	49.06	11.72(5.61)
Gastrointestinal disorders	Pouchitis	5	11.39(4.72-27.5)	11.39(4.72-27.49)	11.28(5.4)	46.91	11.28(5.4)
General disorders and administration site conditions	Infusion site mass	5	8.94(3.71-21.56)	8.94(2.48-14.37)	8.87(4.25)	34.97	8.87(4.25)
Gastrointestinal disorders	Pancreatic mass	5	7.98(3.31-19.23)	7.98(2.64-15.31)	7.92(3.8)	30.28	7.92(3.8)
General disorders and administration site conditions	Early satiety	5	7.27(3.02-17.51)	7.27(8.32-48.79)	7.23(3.46)	26.84	7.23(3.46)
Gastrointestinal disorders	Rectal prolapse	5	7.14(2.96-17.21)	7.14(2.14-12.43)	7.1(3.4)	26.25	7.1(3.4)
Neoplasms benign, malignant and unspecified (incl cysts and polyps)	Acrochordon	5	6.36(2.64-15.31)	6.36(1.87-10.84)	6.33(3.03)	22.44	6.33(3.03)

Gastrointestinal disorders	Intestinal mass	5	5.97(2.48-14.38)	5.97(6.08-35.5)	5.94(2.85)	20.57	5.94(2.85)
Injury, poisoning and procedural complications	Incisional hernia	5	5.79(2.4-13.94)	5.79(3.71-21.55)	5.76(2.76)	19.69	5.76(2.76)
Investigations	Blood culture positive	5	5.4(2.24-13)	5.4(5.31-30.99)	5.38(2.58)	17.83	5.38(2.58)
Gastrointestinal disorders	Intestinal ulcer	5	5.37(2.23-12.92)	5.37(2.02-11.71)	5.35(2.56)	17.68	5.35(2.56)
Neoplasms benign, malignant and unspecified (incl cysts and polyps)	Neuroendocrine tumour	5	5.16(2.15-12.43)	5.16(6.8-39.79)	5.14(2.47)	16.71	5.14(2.47)
Investigations	Serum ferritin decreased	5	5.15(2.14-12.39)	5.14(10.7-63.04)	5.13(2.46)	16.62	5.13(2.46)
Renal and urinary disorders	Prerenal failure	5	4.87(2.02-11.72)	4.87(3.02-17.51)	4.85(2.32)	15.29	4.85(2.32)

Gastrointestinal disorders	Change of bowel habit	5	4.54(1.89-10.94)	4.54(2.96-17.21)	4.53(2.17)	13.76	4.53(2.17)
Investigations	Carcinoembryonic antigen increased	5	4.5(1.87-10.84)	4.5(9.23-54.21)	4.49(2.15)	13.57	4.49(2.15)
